# A Cross-Sectional Time Course of COVID-19 Related Worry, Perceived Stress, and General Anxiety in the Context of Post-Traumatic Stress Disorder-like Symptomatology

**DOI:** 10.3390/ijerph19127178

**Published:** 2022-06-11

**Authors:** Roger J. Mullins, Timothy J. Meeker, Paige M. Vinch, Ingrid K. Tulloch, Mark I. Saffer, Jui-Hong Chien, O. Joseph Bienvenu, Frederick A. Lenz

**Affiliations:** 1 Department of Neurosurgery, Johns Hopkins University, Baltimore, MD 21287, USA; rmullin3@jh.edu (R.J.M.); pvinch1@jhu.edu (P.M.V.); msaffer3@jhmi.edu (M.I.S.); jchien7@jhmi.edu (J.-H.C.); flenz1@jhmi.edu (F.A.L.); 2 Department of Psychology, Morgan State University, Baltimore, MD 21251, USA; ingrid.tulloch@morgan.edu; 3 Department of Psychiatry and Behavioral Sciences, Johns Hopkins University, Baltimore, MD 21287, USA; obienve1@jhmi.edu

**Keywords:** COVID-19, post-traumatic stress disorder, survey, questionnaire, somatization

## Abstract

The COVID-19 pandemic within the United States of America resulted in over 800,000 deaths as of February 2022 and has been addressed by social distancing or stay-at-home measures. Collective prolonged multimodal trauma on this scale is likely to elicit symptomatology in the general population consistent with post-traumatic stress disorder (PTSD), somatization, anxiety, and stress. The psychological component of this response contributes substantially to the burden of this disease worldwide. This cross-sectional study examines the relationship between COVID-19-related concern, anxiety, and perceived stress on PTSD-like symptomatology over the course of the COVID-19 pandemic. Participants were recruited via social media within the United States of America between 8th May 2020 and 11th August 2021 to complete an internet questionnaire including mood, personality, and COVID-19-specific scales. General anxiety and PTSD-like symptomatology were above the screening cutoffs for most respondents. These measures increased in severity over the pandemic, with the change point of our Concern scale preceding that of the other significant measures. Measures of COVID-19-related concern, generalized anxiety, and PTSD-like symptomatology were strongly correlated with each other. Anxiety, perceived stress, and PTSD-like symptomatology are strongly interrelated, increase with pandemic length, and are linked to reported levels of concern over COVID-19. These observations may aid future research and policy as the pandemic continues.

## 1. Introduction

Since the first case of disease from the novel coronavirus, SARS-CoV-2, causing COVID-19, was diagnosed in the United States (US) on 20 January 2020, numerous states and municipalities have instituted various levels of social distancing and stay-at-home measures [1]. As of 1 February 2022, the US CDC reported that there were more than 75 million confirmed positive cases of infection with the novel coronavirus and more than 884,853 deaths. Given the widespread impact of these events, the known ability of collective mass multimodal trauma to induce psychiatric disorders such as generalized anxiety disorder (GAD), post-traumatic stress disorder (PTSD), and comorbid functional pain syndromes, it is likely that there will be a surge in mental health and general health complaints following the COVID-19 pandemic comparable to that of the 9/11 terrorist attacks [2,3,4,5,6,7]. Participants reporting high levels of general anxiety, perceived stress, and somatization, the propensity to report internal body sensations in the terms of possible illness, are at a greater risk for GAD, PTSD, and comorbid functional pain syndromes [6,8,9,10,11,12].

While several studies have examined the effects of collective mass multimodal trauma on PTSD and GAD, the contribution of somatization has rarely been investigated [6,13]. Somatization should be a particularly important factor in a pandemic environment with social distancing and stay-at-home orders, where self-report and perception of relevant symptoms may be heightened. Additionally, during the continuing COVID-19 pandemic, widespread adoption of vaccination in the United States and the slow return of society to normal provided a natural temporal experiment to investigate the temporal evolution of interrelations of perceived stress symptoms, general anxiety, and traumatic-stress-like symptoms.

During the global COVID-19 pandemic, numerous studies have reported strong relationships between measurement scales for anxiety, perceived stress, and post-traumatic symptomatology. Strong correlations between anxiety and perceived stress were revealed by many surveys using the GAD-7 and PSS scales [14,15,16,17,18,19,20,21,22,23]. Anxiety and PTSD-like symptomatology have also shown consistent positive correlations during the pandemic, as measured by the GAD-7 and IES scales [24,25,26]. Similarly, post-traumatic symptomatology relates to perceived stress measured by the PSS and IES scales [27], while somatization has been shown to correlate strongly with anxiety, perceived stress, and post-traumatic symptomatology [28] during the pandemic.

Given the highly intercorrelated nature of the symptoms implied by these measures, one response has been to narrow the focus toward the common feature of concern, fear, or worry specifically related to the COVID-19 pandemic. This self-reported factor holds a strong relation to the prior measures and may have substantial explanatory power [29,30,31]. Some researchers even present the possibility that pandemic-related concern may represent its own type of trauma [32].

The objective of this study was to determine the temporal relationships among general anxiety, perceived stress, COVID-19-related concern, somatization, and post-traumatic stress symptomatology. We hypothesized that the persistent stress of the COVID-19 pandemic would increase perceived stress, followed by generalized anxiety, leading to the expression of post-traumatic stress symptomatology. Our first research question pertained to how general measures of anxiety and perceived stress would relate to more specific measures of somatization, post-traumatic stress symptomatology, and COVID-19-related concern. We predicted a positive relationship between these measures. Our second research question was how these measures would change over the observed course of the COVID-19 pandemic, predicting a general increase over time with peaks reflective of change points when and if the trend diverted. Our third research question involved how COVID-19-related concern would be related to measures of behavior that could lead to SARS-CoV-2 exposure, with our prediction being an inverse relationship.

## 2. Materials and Methods

### 2.1. Survey Participation

Subjects answered questions from standardized psychological questionnaires (see Table 1) as well as a novel COVID-19-specific questionnaire developed specifically for this study. Recruitment took place as advertisements on social media sites such as Twitter, Facebook, Reddit, and institutionally affiliated research websites. Potential participants used a web link to the survey and completed several psychological questionnaires. Because of the nature of the study, the Johns Hopkins School of Medicine Institutional Review Board waived informed consent. Each participant was assigned a participant number to uniquely identify their data and keep protected health information separate from their responses to COVID-19-specific and more general health and psychological surveys. This survey was implemented with a Qualtrics XM account and required approximately 60 to 120 min to complete. Participants were allowed to save their data and continue the survey later to complete it. The first participant completed the survey on 8 May 2020, and it was closed to new responses on 11 August 2021.

### 2.2. Measures

In addition to the independent COVID-19-specific questions (see Supplementary Data), the online survey evaluated anxiety (GAD-7 [33], Anxiety Sensitivity Inventory [34]), PTSD-like symptomatology (Impact of Events Scale-6 [35,36]), somatization (Pennebaker’s Inventory of Limbic Languidness [37]), Somatic Symptom Scale-8 [38], and Somatic Symptom Disorder–B criteria scale [39], depression (PHQ-9 [40]), neuroticism (as inverse of stability on the Big Five Factor Scale [41]), perceived stress (Perceived Stress Scale-14 [42]), health-related quality of life (SF-36 [43]), pain sensitivity (Pain Sensitivity Questionnaire [44]), and sleep quality (Pittsburgh Sleep Quality Inventory [45]). See Table 1 for the psychometric properties of these measures. The start of the pandemic in January 2020 was indicated as the event for the IES-6 scale.

### 2.3. Data Analysis

All analyses were performed in R (version 4.1.0, R Core Team 2021, Vienna, Austria). Questionnaire responses were downloaded and collated using the R QualtRics package (version 3.1.5). A custom R script calculated the derived scores and subscores for each of the assessment scales, based on the scoring methods described in their original sources. Data were excluded if the respondent spent less than two minutes on the survey or did not report a valid US zip code. Sporadic missing data were accounted for within each questionnaire using PCA imputation (R package missMDA, version 1.18), ranging from 0 to 10.4%. Entirely empty rows of data within a questionnaire were not imputed and instead reflected in the number of participants, with such nonresponses ranging from 15.9% to 53.4% of the data as the survey progressed. Simple multiple-choice arithmetic and general knowledge questions were interspersed within the survey to assess attention, such as “4 + 5 =” and “which of the following things is alive.” Responses for these questions were consistently correct throughout the survey and no data were excluded on that basis.

To condense the COVID-19-specific questions into composite scores, we performed a factor analysis of the COVID-19-related questions in R package FactoMiner (version 2.4) using the PCA and FactoInvestigate functions. This PCA analysis was used to evaluate assumptions that specific groups of these questions would reflect issues of concern or worry, levels of estimated exposure, and physical symptoms related to COVID-19.

Age, sex, education, and race were assessed for potential demographic effects on the measures included in the questionnaire. This analysis used a series of linear regressions with age, race, sex, or education as the independent variable and each separate measure as the dependent variable. Higher age was associated with lower PSS-14 totals. Higher age was also associated with higher SF-36 role limitations due to emotional problems, COVID-19-specific questionnaire Concern scale scores, SF-36 energy/fatigue, and Big 5 Stability. Higher education was associated with lower PHQ-9 and PSS totals. There were no significant differences in the questionnaire measures for sex or race after Benjamini–Hochberg (BH) correction for multiple comparisons, but sex was retained as a covariate due to the notably large proportion of female respondents (77%). See Supplementary Table S2 for statistics related to the selection of covariates.

Scale-to-scale comparisons used a partial correlation between each score, with age, sex, and years of education as covariates. The Benjamini–Hochberg procedure was used to adjust resultant *p*-values for multiple comparisons. Tests of the difference between two correlated correlations were performed using the r.test function of the R psych package (version 2.1.9).

We used a general linear model to assess cross-sectional changes in questionnaire responses over the course of the pandemic. The independent variable was time, expressed as the number of days since the start of the study. Scores for the scales used were the dependent variable, with age, sex, and years of education as covariates. The Benjamini–Hochberg procedure was used to adjust the resultant p-values for multiple comparisons. To assess the point in the cross-sectional time series in which the trend “breaks” or changes direction, we also used change point analysis (R changepoint package version 2.2.2) using the At Most One Change (AMOC) algorithm.

## 3. Results

### 3.1. Demographics

**Figure 1:**
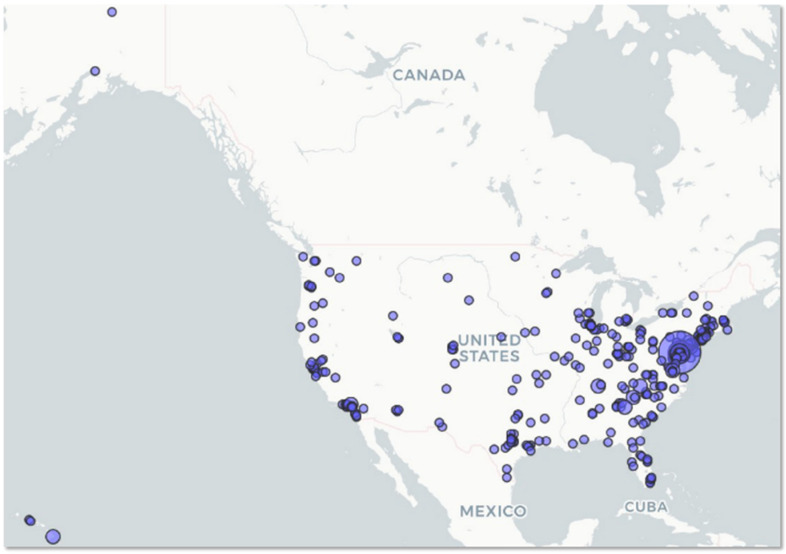
Geographic location of respondents. Figure created in R mapview package; area of circles represents the number of respondents in that US zip code.

General characteristics of the respondents are shown in Table 2, with expanded information available in Supplementary Table S1. Geographic location of respondents is in Figure 1. The sample tended toward respondents who are white (72%), female (77%), possess some college education (29%), or earn income under 40,000 USD per year (39%). All reported valid zip codes located within the United States of America.

### 3.2. COVID-19-Specific Questionnaire

According to the responses to the COVID-19-specific questions in the survey, most respondents stayed home to avoid infection (73%) and started isolation in March 2020 (59.3%). The majority wore a mask when going outside of their home (58.6%) with the most popular choice being a cloth mask (44.1%). A plurality was either a little bit (26.5%) or moderately worried (20.8%) about contracting COVID-19 within the next month. Response breakdowns for other individual COVID-19-specific questions are shown in Supplementary Table S3.

#### Cross-Sectional Analysis of Individual COVID-19-Specific Questions

Some behaviors and attitudes measured by the COVID-19-specific questions changed over the survey period. Over time, there were significant cross-sectional increases in the response values toward the questions “How many people do you live with in your residence?” (F (4, 338) = 15.86, *p* = 1.56 × 10^−10^) and “Are you able to perform most of your job functions working remotely from home?” (F (4, 331) = 8.73, *p* = 0.00002). There were cross-sectional decreases in response values toward the questions “Do you believe that you have a higher risk for serious illness from the novel coronavirus or COVID-19?” (F (4, 322) = 10.32, *p* = 1.62 × 10^−6^), “Considering any chronic diseases you may have, how concerned are you that you may contract the novel coronavirus or COVID-19 in the next month?” (F (4, 322) = 6.31, *p* = 0.0014), “How often are you exercising per week?” (F (4, 335) = 5.08, *p* = 0.01) and “How much news or other COVID-19-related content have you watched each day in the past week?” (F (4, 322) = 4.94, *p* = 0.013). Change point analysis did not reveal any discernible breaks in the trends over the survey period.

### 3.3. Factor Analysis for Subscale Generation

To draw out composite measures of COVID-19-related concern, exposure, and symptomatology, principal component (PCA) factor analysis was performed on a subset of the 24 COVID-19-specific questions that yielded ordinal or continuous outcomes. This revealed two primary dimensions explaining 29.9% of the total inertia of the dataset, with dimension 1 explaining 16.5% and dimension 2 explaining 13.4%. The questions were divided into three scales: Concerns, Exposure, and Symptoms. The PCA factor dimensions and valence (+/−) were used to guide and assess the scale membership of each question. Questions comprising the Concern and Symptoms scales fell largely within the same factor but were retained as separate scales based on prior intent. Four questions were excluded as their factor cosine was less than 0.10 in the highest dimension, leaving a total of 20 questions with explanatory power. The remaining individual responses were then divided by their maximum possible value, reverse scored if their dimension factor was negative, and averaged. For ease of interpretation, we multiplied each fractional average scale by 100 to fit a 1–100 scale. See Table 3 for details. Cronbach alpha scores for the three scales were Concern = 0.79, Exposure = 0.75, and Symptoms = 0.29. Only the more reliable Concern and Exposure scales were carried forward into the following analyses.

### 3.4. Threshold for Anxiety, Depression, and PTSD-like Symptomatology

**Figure 2:**
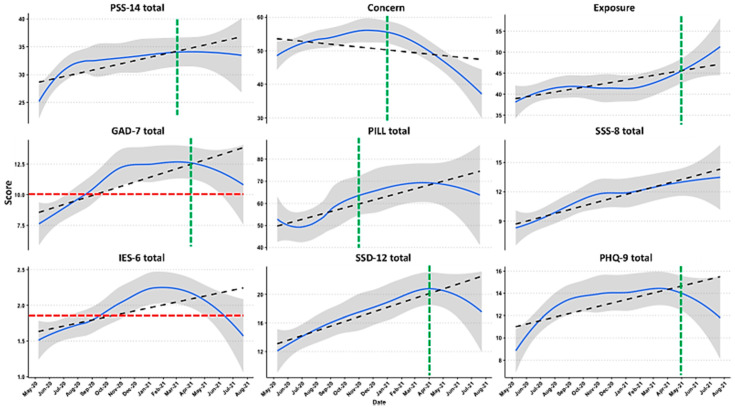
Cross-sectional timeline responses to standard assessment scores and subscores over the course of the pandemic. The top row includes perceived stress (PSS-14) and the derived COVID-19-specific questionnaire scales. The middle row includes measures of anxiety (GAD-7 total) and somatization (PILL, SSS-8). The bottom row is for PTSD-like symptomatology (IES-6), somatization (SSD-12), and depression (PHQ-9). The red dotted horizontal line indicates the screening cutoffs for GAD-7 (10) and IES-6 (1.75), and the green dotted vertical line shows the month and year at which the trend deflects as per the change point analysis. The blue line is loss curve with standard error (se) shading. The black dotted line is a linear model fit (regression line). The figure was created with R ggplot2.

The key standard assessment scales for anxiety and PTSD-like symptomatology include guidelines or cutoff thresholds considered symptomatic for those conditions. The average respondent exceeded these thresholds midway through the pandemic period assessed by the survey. A score of 1.75 on the IES-6 total indicates substantial PTSD-like symptoms [36]. The mean IES-6 total was 1.8 ± 1.0 (95% CI: 1.74–1.96), and this threshold was met or exceeded by the majority (55.8%) of respondents by September 2020. The GAD-7 indicates symptoms of anxiety with a cutoff of 10 [33]. The mean GAD-7 total was 10.4 ± 6.4 (95% CI: 9.69–11.10), with the clinical threshold exceeded by 56.1% of the respondents by September 2020. Timeline plots with cutoffs for the primary measures of interest based on our aims are shown in Figure 2. Descriptive statistics for all assessment scales are found in Table 4, and a complete set of timeline plots for all measures is available as Supplementary Figure S1.

#### 3.4.1. Association between Generalized Anxiety and PTSD-like Symptomatology

**Figure 3:**
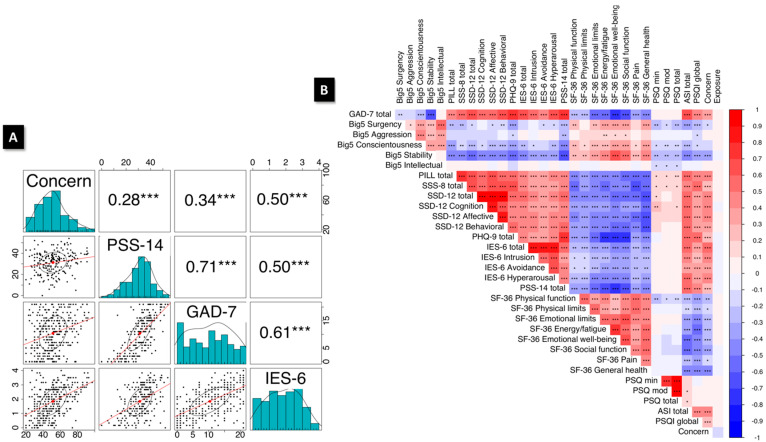
Partial correlations for the survey scales and subscales. (**A**) Lower triangle consists of a scatterplot showing linear regression lines and convex hulls for the PSS-14 total, GAD-7 total, and IES-6 total. Histograms for each measure are shown on the diagonal, and the r values on the upper triangle. (**B**) Correlogram of all survey scales and subscales, with red for positive and blue for negative correlations. Asterisks in A and B represent Benjamini–Hochberg corrected *p*-values: * = *p* < 0.05, ** = *p* < 0.01, *** = *p* < 0.001.

We predicted that general anxiety and perceived stress would positively correlate with somatization, post-traumatic stress symptomatology, and COVID-19-related concern. Partial correlation analysis showed strong positive associations (Figure 3A) between scores for COVID-19-related Concern, generalized anxiety (GAD-7), perceived stress (PSS-14), and PTSD-like symptomatology (IES-6). The entire battery of measures also showed consistent associations among most scales (Figure 3B).

#### 3.4.2. Cross-Sectional Trajectory of Standard and COVID-19-Specific Measures

To assess changes in attitudes and symptomatology over time, the COVID-19 survey included a series of standard assessments for anxiety, somatization, pain sensitivity, stress, and aspects of personality. There were cross-sectional increases in the scores for PSS-14 total, SSS-8 total, GAD-7 total, SSDB total, PHQ-9 total, SSDB affective subscore, SSD-12 behavioral subscore, SSDB cognitive subscore, IES-6 Avoidance, ASI total, and PILL total. There were cross-sectional decreases over time in the scores for SF-36 energy/fatigue, SF-36 social functioning, Big 5 stability, SF-36 role limitations due to emotional problems, and SF-36 role limitations due to physical health. Statistics for these significant scales are available in Table 5, and a complete list of statistics including nonsignificant results is in Supplementary Table S5.

Change point analysis revealed trend deflections in November 2020 for the PILL total, March 2021 for the PSS-14 and ASI total, April 2021 for the GAD-7 total, SSD-12 total, and SF-36 physical health subscales, May 2021 for the PHQ-9 total, and June 2021 for the SF-36 energy/fatigue, SF-36 Social Functioning, Big-5 Stability, SF-36 role limitations due to emotional problems, and PILL total.

Next, we focused on the question of whether COVID-19-related concern would be inversely related to measures of behavior that could lead to SARS-CoV-2 exposure. During the course of the COVID-19 pandemic captured in the survey, only the Concern scale showed a significant decrease over time (F (4, 321) = 6.84, *p* = 0.001). The trends for the Concern and Exposure scales were significantly different from each other via the r test of difference between two correlated correlations (T (1, 312) = −3.32, *p* = 0.001). Change point analysis revealed downward trend changes in Concern starting in January of 2021 and upward changes in exposure starting June of 2021. Timeline plots of selected scales are shown in Figure 2, timeline plots for all measures are shown in Supplementary Figure S1, and statistics for each of the standard assessment scales are in Supplementary Table S4.

## 4. Discussion

This study focused on the related factors of anxiety, perceived stress, somatization, PTSD-like symptoms, and COVID-19-related concern during the COVID-19 pandemic in the United States of America. Over the course of the pandemic from 8 May 2020 to 11 August 2021, we found compelling spikes in these measures matching those found in other studies during this time period [29,31,46,47,48,49,50,51]. In our cross-sectional analysis, many of these symptoms increased over time, adding a sense of urgency to the understanding that the COVID-19 pandemic has had a deleterious effect on worldwide mental health.

As predicted, we found strong positive correlations between the GAD-7, PSS-14, and IES-6 measures—representing anxiety, perceived stress, and PTSD-like symptoms. These interrelated factors also correlated well with a novel measurement of COVID-19-related concern, lending credence to the idea that these factors are related. No directional component could be inferred from this analysis, partly due to the cross-sectional nature of the measures.

Interestingly, COVID-19 Concern scores decreased during the course of the pandemic as self-reported exposure to potential risks increased. This observation is consistent with the widespread notion of attempting to go “back to normal,” especially after vaccines became available. Measures of anxiety, stress, somatization, and PTSD also correlated strongly with Concern scores, providing some hope that this pattern will reverse itself if and when the threat of COVID-19 recedes.

Change point analysis was used to identify points in the timeline where assessment scores broke or deflected. Our prediction was that perceived stress (PSS-14) scores would change first, followed by general anxiety (GAD-7), then post-traumatic stress disorder-like symptomatology (IES-6). This was borne out in the resulting data which showed the PSS-14 peaking on March 2021, GAD-7 peaking on April 2021, and the IES-6 showing no detectable peak by change point analysis (Figure 2). While the visually discernible peak for IES-6 does seem to follow the trend, this scale either may use too few units within the measure to show fine changes or simply had not actually reached a true change point at the time the survey ended. The COVID-19-related Concern score was by far the earliest indicator, showing downward deflection in January 2021. A salient time point for comparison is December 2020, the month at which COVID-19 vaccines became available. This analysis does not imply causality of one event or measure upon another but does yield insight into the duration of pandemic-related symptomatology.

The relationship between somatic and PTSD-like symptoms has long been evident among veterans with PTSD [52,53,54]. We found strong positive correlations of somatic symptoms and somatization with COVID-19 Concern scores as well as PTSD-like symptoms in the present study. Interestingly, while somatization as measured by the PILL, SS8, and somatic symptoms as measured by the SSD-12 scale all loaded on the same factor during principal component analysis, indicating a redundancy of information, somatization as measured by the PILL showed a significant change in trajectory in November 2020 and reached its highest point during February and March 2021; the SSD-12 scale of somatic symptoms peaked and demonstrated a change in trajectory in April 2021. Future studies are needed to test whether this was a seasonal effect related to allergies or seasonal affective disorder, since many symptoms listed on the PILL are elevated during bouts of seasonal allergies and may be elevated during seasonal affective disorder [37]. Recent studies have found that elevated somatization in PTSD is related to overall lifetime trauma more strongly than combat exposure and that somatic disorder is present in nearly 60 percent of those with probable PTSD compared to 5 percent of those free of PTSD symptoms [55]. Furthermore, patients with complex PTSD, which is more often a result of trauma exposure of varying types and durations, report greater somatization on the PHQ-15 compared to those with simple PTSD [56]. This study found the relationship between PTSD and somatization is probably bidirectional and mutually maintaining, while the maintenance of PTSD and somatization can still be seen more than 20 years after the events which triggered PTSD [56,57]. Finally, our findings of clinically significant levels of somatization in a large proportion of the general population during the COVID-19 pandemic are consistent with previously published studies in the general population and in healthcare professionals [58,59,60,61]. These factors, combined with the high burden of PTSD-like symptoms coupled with somatization in our study, suggest this psychological burden may persist for years following the COVID-19 pandemic.

Limitations of this study include the characteristics of the sample and psychometric features of the COVID-19-specific questions. Unavoidably, respondents were drawn from a sample of those with access to the internet and social media. As participation was not compensated, the sample of respondents may also be skewed toward those with the time or disposition to fully complete the survey without tangible reward. The sex of the respondents also skewed heavily toward female respondents. As for the psychometric features of the COVID-19-specific scales, the current version of the COVID-19-specific subscores for concern, exposure, and symptoms is not designed for hand scoring and is reliant on an automated script. Future revisions to this scale will include regularizing the range of the number of responses within questions to ease manual scoring and the removal of exploratory questions that explained little variance in the responses.

## 5. Conclusions

Using over a year of online survey data during the COVID-19 pandemic, we found that anxiety, perceived stress, and PTSD-like symptomatology are strongly interrelated, increase with pandemic length, and are strongly linked to reported levels of concern over COVID-19. Further, change point analyses showed peaks for concern preceding other measures, as well as showing downward deflections in symptomatology after the availability of vaccinations. Our results strengthen support for the hypothesis that the trajectory of risk factors leading to PTSD-like symptoms begins with enhanced general distress coupled with concern or worry about the initiating stressful life events. The initial events, which may threaten life or way of life, snowball into pathological distress, generalized anxiety, and PTSD-like symptomatology. Particularly relevant in the case of a public health disaster but clearly relevant in other etiologies of PTSD, somatization and anxiety sensitivity seem associated with individuals experiencing a greater PTSD-like symptom burden [52,53,57,62,63,64]. Our current study and other cross-sectional results implicate somatization as a risk factor in the development of PTSD. This may help to explain the high comorbidity between chronic pain disorders and PTSD as somatization is risk factor for both disorders [65,66,67,68].

As the pandemic is ongoing, the observations from this study will help inform and guide future research and public policy on this overwhelmingly important worldwide phenomenon. Since greater reports of somatization and anxiety sensitivity are associated with development and severity of PTSD-like symptomatology, these individuals should be prioritized for early intervention during and after disasters. Effective modes of intervention may include early psychological first aid, and traditional group and one-on-one cognitive behavioral therapy (CBT) [69,70]. Business managers, education and healthcare professionals, and others in a supervisory role should be educated in the signs of enhanced perceived stress, somatization and psychological first aid. Resources for CBT and mental health care must be destigmatized, and their availability broadened to ensure those susceptible to PTSD have the resources to remain resilient. Training and the application of psychological first aid and referral to mental health resources should be provided as in-depth programs rather than trivial exercises in regulatory satisfaction [70]. This may be especially necessary in educators, as younger participants seem to experience a greater burden of symptoms that are associated with the development of PTSD [30,62,71,72,73,74,75,76,77,78].

## Figures and Tables

**Table 1:** Psychometric properties of standard scales.

Name	Items	Item Range	Scores	Score Range	Cronbach’s α
GAD-7	7	0–3	Anxiety	0–21	0.92
ASI	16	0–4	Anxiety sensitivity	0–64	0.91
PHQ-9	9	0–3	Depression	0–27	0.90
SF-36	36	0–100 (extrapolated)	Physical functioning Physical health Emotional problems Energy/fatigue Emotional well-being Social functioning Pain general health	0–100 0–100 0–100 0–100 0–100 0–100 0–100 0–100	0.93 0.88 0.86 0.83 0.85 0.86 0.85 0.83
Big 5	100	1–5	Surgency Agreeableness Conscientiousness Stability Intellectual	10–100 10–100 10–100 10–100 10–100	0.92 0.90 0.91 0.90 0.89
PSQ	17	0–10	Total pain sensitivity Moderate pain sensitivity Minor pain sensitivity	0–140 0–70 0–70	0.93 0.91 0.87
PSS-14	14	0–4	Perceived stress total	0–56	0.89
IES-6	6	0–4	PTSD-like symptomatology Intrusion Avoidance Hyperarousal	0–4 (mean) 0–4 (mean) 0–4 (mean) 0–4 (mean)	0.86 0.84 0.71 0.76
PSQI	7	0–3	Global sleep quality	0–21	0.74
PILL	54	0–4	Somatization total	0–216	0.94
SSS-8	8	0–4	Somatization total	0–32	0.80
SSD-12	12	0–4	Somatization total Cognitive Affective Behavioral	0–48 0–16 0–16 0–16	0.94 0.74 0.86 0.91

**Table 2:** General characteristics of respondents.

Demographic	N (%) or Mean ± SD
Total participants	408
Age in years (range 17–85)	34.1 ± 13.11
Sex	
Female	314(77%)
Intersex	1(0.2%)
Male	91(22.3%)
Race	
White	293(71.8%)
Native Hawaiian or Other Pacific Islander	0(0%)
Black or African	39(9.6%)
Asian American	28(6.9%)
American Indian or Alaska Native	2(0.5%)
Other	33(8.1%)
Refuse	4(1%)
Do Not Know	7(1.7%)

**Table 3:** Factor analysis for COVID-specific subscales.

Q#	Dim.1	Dim.2	Scale	Question (Paraphrased)
61	−0.36	0.16	Concern	Wearing a mask when going outside.
81	0.75	0.15	Concern	Worry about contracting COVID-19 in the next month.
82	0.75	−0.12	Concern	Concern about leaving residence due to COVID-19.
84	0.44	−0.23	Concern	Frequency of watching news about COVID-19.
85	0.74	−0.05	Concern	Concern over chronic disease-related COVID-19 vulnerability.
86	0.59	−0.02	Concern	Perceived risk for serious COVID-19-related illness.
54	−0.32	0.56	Exposure	Stay home to avoid infection.
58	−0.24	0.66	Exposure	People within 6 feet.
59	−0.19	0.62	Exposure	People in physical contact.
60	−0.29	0.67	Exposure	Left place of residence last week.
63	0.06	−0.66	Exposure	Employed as Essential Worker.
64	0.13	0.54	Exposure	Able to perform job remotely.
80	−0.32	−0.52	Exposure	Contact with COVID-19-infected person in past week.
70	0.27	0.44	Symptoms	Taking temperature in the past week.
71	0.48	0.19	Symptoms	Cough in the past week.
72	0.52	0.22	Symptoms	Breathing trouble in the past week.
74	0.37	0.14	Symptoms	Chills in the past week.
75	0.34	0.18	Symptoms	Lost sense of smell in the past month.
78	0.38	0.15	Symptoms	Consulted Doctor about COVID-19 in past month.
79	−0.36	−0.33	Symptoms	Contracted COVID-19 in the past month.

Note: Q# is question number, Dim. is the dimension number (1 or 2).

**Table 4:** Descriptive statistics for questionnaire measures.

Measure	N	Mean ± SD	95% CI	MAR
COVID-19-Specific Questionnaire:				
Concern	347	51.37 ± 15.84	49.7–53.04	4.1%
Exposure	347	41.91 ± 14.46	40.38–43.43	1.2%
Symptoms	347	34.56 ± 6.75	33.84–35.27	2.1%
Anxiety:				
GAD-7 total	319	10.39 ± 6.4	9.69–11.1	0.6%
ASI total	213	19.88 ± 13.69	18.03–21.73	0.4%
Personality/Neuroticism:				
Big 5 Surgency	213	100.17 ± 28.4	96.34–104.01	1.6%
Big 5 Agreeableness	213	132.11 ± 22.81	129.03–135.19	2.0%
Big 5 Conscientiousness	213	123.28 ± 24.21	120.01–126.55	2.0%
Big 5 Emotional Stability	213	90.75 ± 26.09	87.22–94.27	1.7%
Big 5 Intellect	213	133.56 ± 22.8	130.48–136.64	1.9%
Somatization:				
PILL total	213	57.44 ± 32.68	53.03–61.86	0.9%
SSS-8 total	319	10.66 ± 6.47	9.95–11.37	0.9%
SSD-12 total	319	16.39 ± 11.09	15.17–17.61	0.1%
SSD-12 cognition	319	5.13 ± 3.51	4.75–5.52	0.2%
SSD-12 affective	319	6.16 ± 4.08	5.71–6.61	0.0%
SSD-12 behavioral	319	5.31 ± 4.45	4.82–5.8	0.1%
Depression:				
PHQ-9 total	313	12.57 ± 7.13	11.77–13.36	1.0%
PTSD-like Symptomatology:				
IES-6 total	330	1.85 ± 1	1.74–1.96	0.1%
IES-6 Intrusion	330	1.82 ± 1.12	1.7–1.94	0.2%
IES-6 Avoidance	330	1.71 ± 1.13	1.58–1.83	0.0%
IES-6 Hyperarousal	330	2.02 ± 1.26	1.88–2.15	0.2%
Perceived Stress:				
PSS-14 total	214	31.21 ± 9.93	29.87–32.55	0.3%
General Health:				
SF-36 Physical Functioning	214	84.25 ± 22.55	81.21–87.29	1.7%
SF-36 Physical Role Limitations	214	62.97 ± 41.16	57.42–68.51	2.1%
SF-36 Emotional Role Limitations	214	31.91 ± 41.17	26.36–37.46	2.5%
SF-36 Energy/Fatigue	214	31.64 ± 21.84	28.69–34.58	1.8%
SF-36 Emotional Well-being	214	45.2 ± 23.53	42.03–48.37	1.6%
SF-36 Social Functioning	214	54.06 ± 33.04	49.61–58.51	2.3%
SF-36 Pain	214	71.41 ± 24.11	68.16–74.66	1.2%
SF-36 General Health	214	55.93 ± 22.8	52.86–59.01	0.7%
Pain Sensitivity:				
PSQ minimal	212	19.04 ± 10.54	17.62–20.47	0.4%
PSQ moderate	212	31.35 ± 12.28	29.69–33.02	0.5%
PSQ total	212	50.4 ± 21.5	47.49–53.31	0.4%
Sleep Quality:				
PSQI Global score	197	9.23 ± 4.4	8.61–9.85	2.6%

Note: CI is confidence interval (lower–upper), MAR is missing-at-random.

**Table 5:** Linear model results for assessment scales during the COVID-19 pandemic.

Scale	df	F	*p*-Value (BH)	Change Point
PSS-14 total	4, 207	10.98	0.00000	March 2021
SSS-8 total	4, 311	9.36	0.00001	NA
SF-36 energy/fatigue	4, 207	8.12	0.00013	June 2021
GAD-7 total	4, 311	7.72	0.00018	April 2021
SSD-12 behavioral	4, 311	6.72	0.00097	NA
Concern	4, 338	6.69	0.00119	January 2021
SF-36 social functioning	4, 207	6.68	0.00097	June 2021
SSD-12 total	4, 311	6.54	0.00119	April 2021
PHQ-9 total	4, 305	6.14	0.00263	May 2021
Big 5 stability	4, 206	6.11	0.00243	June 2021
SSD-12 cognitive	4, 311	5.99	0.00270	NA
SSD-12 affective	4, 311	5.87	0.00319	NA
SF-36 emotional role limitations	4, 207	5.67	0.00498	June 2021
IES-6Avoidance	4, 321	4.84	0.01684	NA
PILL total	4, 206	4.52	0.02999	November 2020
SF-36 physical role limitations	4, 207	4.50	0.02999	April 2021
ASI total	4, 206	4.16	0.04965	March 2021

Note: Df is degrees of freedom (numerator, denominator), F is F-value, BH is Benjamini–Hochberg correction for multiple comparisons.

## Data Availability

The data that support the findings of this study are available on request from the corresponding author. The data are not publicly available due to privacy or ethical restrictions.

## Supplementary Materials

The following supporting information can be downloaded at: https://www.mdpi.com/article/10.3390/ijerph19127178/s1.
