# Supplementary material for: A Cross-Sectional Time Course of COVID-19 Related Worry, Perceived Stress, and General Anxiety in the Context of Post-Traumatic Stress Disorder-like Symptomatology
Source: Int J Environ Res Public Health. 2022 Jun 11;19(12):7178. doi: 10.3390/ijerph19127178 (PMC9222603; doi:10.3390/ijerph19127178)
Supplement: Supplementary file 1 [file ijerph-19-07178-s001.zip › Supplementary Tables.pdf]

1 Supplemental

Supplemental Table S1. Expanded characteristics of respondents (N = 408)

Demographic	N (%) or Mean ± SD
Age, yr (range 17-85)	34.1 ± 13.11
Sex:	
Female	314(77)
Intersexed	1(0.2)
Male	91(22.3)
Sexual Identity:	
Heterosexual Male	80(19.6)
Heterosexual Female	215(52.7)
Homosexual Male	8(2)
Homosexual Female	23(5.6)
Asexual	12(2.9)
Bisexual	65(15.9)
Gender Identity:	
Male / Cis Male	78(19.1)
Female / Cis Female	252(61.8)
Trans Male / Trans Man	4(1)
Trans Female / Trans Woman	1(0.2)
Genderqueer / Gender-nonconforming	9(2.2)
Other	4(1)
Race:	
White	293(71.8)
Native Hawaiian or Other Pacific Islander	0(0)
Black or African	39(9.6)
Asian American	28(6.9)
American Indian or Alaska Native	2(0.5)
Other	33(8.1)
Refuse	4(1)
Don’t Know	7(1.7)
Ethnicity:	
Hispanic or Latino	39(9.6)
Not Hispanic or Latino	355(87)
Don't Know	7(1.7)
Refuse	4(1)
Education:	
Less than 8 years	4(1)
8-11 years	13(3.2)
12 years or completed high school	50(12.3)
Post high school training other than college	17(4.2)
Some college	118(28.9)
College graduate	113(27.7)

Post graduate level	53(13)
Professional Degree	26(6.4)
Research Doctorate	13(3.2)
Refuse	1(0.2)
Income:	
$0-$19,999	77(18.9)
$20,000-$39,999	77(18.9)
$40,000-$59,999	53(13)
$60,000-$79,999	45(11)
$80,000-$99,999	28(6.9)
$100,000-$149,999	45(11)
$150,000-$199,999	29(7.1)
$200,000-$249,999	14(3.4)
$250,000-$299,999	5(1.2)
$300,000 or higher	7(1.7)
Refuse	11(2.7)
Marital Status:	
Never married	209(51.2)
Married	110(27)
Living as married	25(6.1)
Divorced	44(10.8)
Separated	8(2)
Widowed	8(2)
Refused	2(0.5)
Work Status:	
Employed	235(57.6)
Unemployed	127(31.1)
Retired	15(3.7)
Disabled	30(7.4)
Financial Standing:	()
Extremely satisfied	41(10)
Moderately satisfied	99(24.3)
Slightly satisfied	39(9.6)
Neither satisfied nor dissatisfied	35(8.6)
Slightly dissatisfied	63(15.4)
Moderately dissatisfied	58(14.2)
Extremely dissatisfied	73(17.9)
Material Standing:	
Extremely satisfied	83(20.3)
Moderately satisfied	116(28.4)
Slightly satisfied	53(13)
Neither satisfied nor dissatisfied	57(14)
Slightly dissatisfied	50(12.3)
Moderately dissatisfied	27(6.6)
Extremely dissatisfied	20(4.9)
Taking Prescription Medications:	

Yes	239(58.6)
No	134(32.8)
Taking Over the Counter Medications:	
Yes	150(36.8)
No	209(51.2)
Smoking:	
Yes	69(16.9)
No	291(71.3)
Drinking:	
Yes	188(46.1)
No	169(41.4)
Caffeine Intake:	
Yes	303(74.3)
No	53(13)

Supplemental Table S1. Individual answers for demographic questions are broken down by response type and described as Number and Percentage (out of 48). NA or missing responses were not included.

Supplemental Table S2. Assessment of Potential Covariates.

Covariate	df1	df2	F	r^2	BH p-value
AGE (years):					
PSS total	1	212	27.90	0.11	0.00001
SF-36 emotional problems	1	212	18.83	0.08	0.00089
Concern	1	345	17.47	0.05	0.00145
SF-36 energy/fatigue	1	212	15.88	0.07	0.00353
Big5 Stability	1	211	13.43	0.06	0.01156
EDUCATION (years):					
PHQ-9 total	1	311	14.64	0.04	0.00645
PSS total	1	212	11.55	0.05	0.03243

Supplemental Table S2. Assessment of Potential Covariates. the BH p-value column is the p-value corrected for multiple comparisons with the Benjamini-Hochberg procedure. Other covariates assessed included sex and race, which yielded no significant differences by the linear model described in the methods section.

Supplemental Table S3. COVID-19 related characteristics of participants (N=408)

Question (paraphrased)	N (%) or Mean ± SD
Stay home to avoid infection	
Yes	298(73)
No	49(12)
Social Distancing Start Date	
Before March 2020	21(5.1)
During March 2020	242(59.3)
After March 2020	64(15.7)
Number of people in residence (Range 1-11)	3.3 ± 1.8
Number of pets in residence (Range 1-11)	2.5 ± 1.7
People within 6 feet	
0	57(14)
1	37(9.1)
2	55(13.5)
3-5	81(19.9)
6-10	31(7.6)
11-20	35(8.6)
21-50	19(4.7)
more than 50	32(7.8)
People in physical contact	
0	157(38.5)
1	45(11)
2	44(10.8)
3-5	60(14.7)
6-10	17(4.2)
11-20	8(2)
21-50	8(2)
more than 50	7(1.7)
Left place of residence last week	
none	48(11.8)
1-3 times	169(41.4)
4-8 times	80(19.6)
9-12 times	24(5.9)
13 or more times	25(6.1)

Wearing a mask when going outside	
Always	239(58.6)
Most of the time	77(18.9)
About half of the time	9(2.2)
Some of the time	15(3.7)
Never	7(1.7)
Mask type worn	
N95 respirator	18(4.4)
Medical mask	128(31.4)
Cloth mask	180(44.1)
Scarf or bandana	15(3.7)
None	6(1.5)
Employed as Essential Worker	
Yes, daily.	38(9.3)
Yes, 3 to 5 times per week.	39(9.6)
Yes, once or twice per week.	12(2.9)
No	258(63.2)
Able to perform job remotely	
Yes	192(47.1)
No	146(35.8)
Worried about COVID-19 exposure	
Not at all	16(3.9)
A little bit	23(5.6)
Moderately	13(3.2)
Quite a bit	14(3.4)
Extremely	22(5.4)
Frequency of exercise	
Never	89(21.8)
Once or twice a week	140(34.3)
3 to 5 times a week	77(18.9)
Daily	37(9.1)
Taking temperature in the past week	
No	202(49.5)
once or twice	72(17.6)
3 to 5 times	29(7.1)
Daily	41(10)
cough in the past week	
No	229(56.1)

once or twice	70(17.2)
3 to 5 times	21(5.1)
Daily	9(2.2)
A few times per day	9(2.2)
Persistent	6(1.5)
Breathing trouble in the past week	
No	264(64.7)
once or twice	51(12.5)
3 to 5 times	15(3.7)
Daily	6(1.5)
A few times per day	3(0.7)
Persistent	5(1.2)
Fever in the past week	
No	337(82.6)
once or twice	4(1)
3 to 5 times	2(0.5)
Daily	0(0)
A few times per day	0(0)
Persistent	1(0.2)
Chills in the past week	
No	300(73.5)
once or twice	28(6.9)
3 to 5 times	12(2.9)
Daily	1(0.2)
A few times per day	2(0.5)
Persistent	0(0)
Lost sense of smell in the past month	
No	329(80.6)
On one or two days	8(2)
Weekly	2(0.5)
Daily	1(0.2)
Persistent	4(1)
Tested for COVID-19 in past month	
Yes	78(19.1)
No	266(65.2)
Positive COVID-19 test in past month	
Yes	6(1.5)
No	67(16.4)

Consulted Doctor about COVID-19 in past month	
No	290(71.1)
Once or twice	37(9.1)
Weekly	1(0.2)
A couple of times per week	2(0.5)
Daily	0(0)
Contracted COVID-19 in the past month	
Definitely yes	4(1)
Probably yes	19(4.7)
Probably not	155(38)
Definitely not	152(37.3)
Contact with COVID-19 infected person in past week	
Definitely yes	14(3.4)
Probably yes	42(10.3)
Probably not	147(36)
Definitely not	127(31.1)
Worry about contracting COVID-19 in the next month	
Not at all worried	70(17.2)
A little bit worried	108(26.5)
Moderately worried	85(20.8)
Quite a bit worried	33(8.1)
Extremely worried	33(8.1)
Concerned about leaving residence due to COVID-19	
Not at all concerned	53(13)
A little bit concerned	88(21.6)
Moderately concerned	86(21.1)
Quite a bit concerned	60(14.7)
Extremely concerned	43(10.5)
Frequency of watching news about COVID-19	
None	61(15)
One or two hours per week	113(27.7)
3 to 5 hours per week	57(14)
About one hour per day	66(16.2)
2 to 5 hours a day	23(5.6)
Most hours of the day	10(2.5)
Concern over chronic disease-related COVID-19 vulnerability	
Not at all concerned or have no chronic diseases	141(34.6)
A little bit concerned	73(17.9)

Moderately concerned	59(14.5)
Quite a bit concerned	30(7.4)
Extremely concerned	27(6.6)
At risk for serious COVID-19 related illness	
No	191(46.8)
Yes, at a slightly increased risk	80(19.6)
Yes, at a moderately increased risk	39(9.6)
Yes, at a severely increased risk	20(4.9)

Supplemental Table S3. Individual answers for the COVID-19 specific questionnaire are broken down by response type and described as Number and Percentage (out of 408). NA or missing responses were not included.

Supplemental Table S4

Question	df1	df2	F	p(unc)	p(bh)	Question Text
Q54	4	339	15.86	6.52E-12	1.56E-10	Have you been staying at home to avoid infection from the novel coronavirus or COVID-19?
Q56	4	338	10.32	7.06E-08	1.62E-06	How many people do you live with in your residence?
Q57	4	337	8.72	1.05E-06	2.30E-05	How many pets do you have living with you?
Q58	4	339	6.31	6.71E-05	0.001	About how many people, outside of your immediate household, have you been within 6 feet of in the past week?
Q59	4	338	5.08	5.50E-04	0.011	About how many people, outside of your immediate household, have you been in physical contact within the past week?
Q60	4	338	4.94	7.08E-04	0.014	About how often within the last week have you left your place of residence within the last week aside from exercise or walks on your own property?
Q61	4	339	3.78	0.005	0.092	How often do you wear a mask when you go outside to locations other than your place of residence?
Q63	4	339	3.41	0.009	0.161	Are you employed in an essential job that you travel to more than twice a week?
Q64	4	331	3.29	0.012	0.183	Are you able to perform most of your job functions working remotely from home?
Q68	4	335	3.14	0.015	0.221	How often are you exercising per week?
Q70	4	336	3.05	0.017	0.240	Have you taken your temperature within the past week?
Q71	4	336	2.60	0.036	0.470	How often have you had a cough in the past week?
Q72	4	336	2.36	0.053	0.597	How often have you been short of breath or had trouble breathing in the past week?
Q73	4	336	2.35	0.054	0.597	Have you had a fever in the past week?
Q74	4	335	2.02	0.092	0.917	How often have you experienced chills in the past week?
Q75	4	336	1.53	0.193	0.925	Have you lost your sense of smell in the last month?
Q78	4	322	1.57	0.183	0.925	Have you spoken with a doctor in a professional capacity (had a consult) about symptoms you believe to be related to the novel coronavirus or COVID-19 this past month?

Q79	4	322	1.56	0.184	0.925	Whether or not you have been tested for the coronavirus or COVID-19, do you believe that you have contracted the coronavirus or COVID-19 in the past month?
Q80	4	322	1.40	0.233	0.925	Do you believe you have been in contact with someone who has the novel coronavirus or COVID-19 in the past week?
Q81	4	321	0.22	0.925	0.925	How worried are you that you may contract the coronavirus or COVID-19 in the next month?
Q82	4	322	1.72	0.144	0.925	How concerned are you to go outside of your place of residence regarding your risk of infection with the novel coronavirus or COVID-19?
Q84	4	322	0.61	0.655	0.925	How much news or other COVID-19 related content have you watched each day in the past week?
Q85	4	322	0.92	0.451	0.925	Considering any chronic diseases you may have, how concerned are you that you may contract the novel coronavirus or COVID-19 in the next month?
Q86	4	322	1.47	0.212	0.925	Do you believe that you have a higher risk for serious illness from the novel coronavirus or COVID-19?

Supplemental Table S4: the p(bh) column is the p-value corrected for multiple comparisons with the Benjamini-Hochberg procedure.

Supplemental Table S5

Measure	DF	F	p-value(bh)	Change
PSS-14 total	4, 207	10.98	0.00000	Mar-21
SSD-12 affective	4, 311	9.36	0.00001	NA
SF-36 emotional role limits	4, 207	8.12	0.00013	Jun-21
GAD-7 total	4, 311	7.72	0.00018	Apr-21
SSD-12 total	4, 311	6.72	0.00097	NA
SF-36 social functioning	4, 207	6.69	0.00119	Jun-21
Concern	4, 338	6.68	0.00097	Jan-21
SSS-8 total	4, 311	6.54	0.00119	Apr-21
Big5 intellect	4, 206	6.14	0.00263	Jun-21
PHQ-9 total	4, 305	6.11	0.00243	May-21
SSD-12 behavioral	4, 311	5.99	0.00270	NA
SSD-12 cognitive	4, 311	5.87	0.00319	NA
SF-36 emotional well being	4, 207	5.67	0.00498	Jun-21
IES-6 total	4, 321	4.84	0.01684	NA
PILL total	4, 206	4.52	0.02999	Nov-20
SF-36 physical role limits	4, 207	4.50	0.02999	Apr-21
ASI total	4, 206	4.16	0.04965	Mar-21
IES6 Intrusion	4, 321	3.92	0.06401	NA
SF-36 pain	4, 207	3.83	0.07592	Jun-21
SF-36 physical functioning	4, 207	3.75	0.08071	May-21
SF-36 energy/fatigue	4, 207	3.70	0.08136	Jun-21
PSQ moderate	4, 205	3.40	0.09745	Jun-21
PSQI global	4, 205	3.38	0.09745	Jun-21
PSQ minimal	4, 190	3.33	0.09745	NA
Exposure	4, 338	3.32	0.09745	May-21
Big5 aggression	4, 206	3.29	0.09745	Jun-21
PSQ total	4, 205	2.71	0.20898	Apr-21
IES-6Avoidance	4, 321	2.62	0.20898	NA
IES6 Hyperarousal	4, 321	2.46	0.22375	NA
SF-36 general health	4, 207	2.34	0.22375	Jun-21
Big5 conscientiousness	4, 206	1.96	0.30763	NA
Big5 surgency	4, 206	1.33	0.35614	Jun-21
Big 5 stability	4, 206	1.10	0.35614	NA

Supplemental Table S5: DF is degrees of freedom (numerator, denominator). The p-value(bh) column is the p-value corrected for multiple comparisons with the Benjamini-Hochberg

procedure. Change is the month at which a break or change in the linear trend took place for that measure.
