# Supplementary material for: A Cross-Sectional Time Course of COVID-19 Related Worry, Perceived Stress, and General Anxiety in the Context of Post-Traumatic Stress Disorder-like Symptomatology
Source: Int J Environ Res Public Health. 2022 Jun 11;19(12):7178. doi: 10.3390/ijerph19127178 (PMC9222603; doi:10.3390/ijerph19127178)
Supplement: Supplementary file 1 [file ijerph-19-07178-s001.zip › Supplementary_COVID19questionnaire.pdf]

Qualtrics Surveys Question Demo

Please click here to review a pdf of the consent document to learn about the study we are inviting you to take part in.

Please sign below to confirm that you have reviewed the online consent document.

× SIGN HERE

clear

Please type your complete name as signed above:

Please type today's date:

We'll start with some demographics questions:

What United States Zip code do you currently reside in?

What sex were you born as?

- Male
- Female
- Intersexed

What is your sexual identity?

- Heterosexual Male
- Heterosexual Female
- Homosexual Male
- Homosexual Female
- Asexual
- Bisexual

What is your current gender identity?

- Male / Cis Male
- Female / Cis Female
- Trans Male / Trans Man
- Trans Female / Trans Woman
- Genderqueer / Gender-nonconforming
- Other

If other, please describe:

How many years old are you?

What is your date of birth?

What is your marital status?

- Never married
- Married
- Living as married
- Divorced
- Separated
- Widowed
- Refused

What is your race?

- White
- Native Hawaiian or Other Pacific Islander
- Black or African
- Asian American
- American Indian or Alaska Native
- ______ Other
- Refuse
- Don't Know

What is your mother's race?

- White
- Native Hawaiian or Other Pacific Islander
- Black or African
- Asian American
- American Indian or Alaska Native
- ______ Other
- Refuse
- Don't Know

What is your father's race?

- White
- Native Hawaiian or Other Pacific Islander
- Black or African
- Asian American
- American Indian or Alaska Native
- ________ Other
- Refuse
- Don't Know

What is 6 + 4?

- 4
- 10
- 16

What is your ethnicity?

- Hispanic or Latino
- Not Hispanic or Latino
- Don't Know
- Refuse

What is your mother's ethnicity?

- Hispanic or Latino
- Not Hispanic or Latino
- Don't Know
- Refuse

What is your father's ethnicity?

- Hispanic or Latino
- Not Hispanic or Latino
- Don't Know
- Refuse

What is the first language you learned to speak?

- English
- Spanish
- [ ] Other
- Refuse

How many years have you lived in the United States?

- All my life
- [ ] Answer in years:

May we email study reminders or forms to you?

- Yes
- No

Please enter your email address:

[ ]

What is the highest grade or level of schooling that you have completed?

[⌄]

What is your current annual household income? Please include all sources of income for all family members who live with you such as wages, salaries, investments, etc.

Overall, how satisfied are you with your current financial situation?

- Extremely satisfied
- Moderately satisfied
- Slightly satisfied
- Neither satisfied nor dissatisfied
- Slightly dissatisfied
- Moderately dissatisfied
- Extremely dissatisfied

Overall, how satisfied are you with the material standards of your life?

- Extremely satisfied
- Moderately satisfied
- Slightly satisfied
- Neither satisfied nor dissatisfied
- Slightly dissatisfied
- Moderately dissatisfied
- Extremely dissatisfied

What is your work status?

- Employed
- Unemployed
- Retired
- Disabled

What is your occupation?

[⌄]

Are you covered by health insurance or some other kind of health care plan? (Please include health insurance obtained through employment or purchased directly as well as government programs like Medicare and Medicaid that provide medical care or help pay medical bills.)

- Yes
- No

What kind of health insurance or health care coverage do you have?

- Private health insurance plan through employer or workplace
- Private health insurance plan purchased directly
- Private health insurance plan through a state, local government, or community program
- Medicare
- Medi-gap
- Medicaid
- CHIP (Children's Health Insurance Program)
- Military health care or VA
- CHAMPUS / TRICARE / CHAMP-VA
- Indian Health Service

For the next part of the survey we will ask you questions about your health and health-related behaviors. Some of these questions may be of a personal nature. Please remember that your answers will be kept confidential and data will be kept as secure as possible.

Have you experienced any of the following health problems in the past 3 months?

	Yes	No
Addison’s Disease	○	○
Adult Attention Deficit Hyperactivity Disorder	○	○
Agoraphobia	○	○
Alcoholism	○	○
Allergies	○	○
Alzheimer's Disease	○	○
Amyotropic Lateral Sclerosis (ALS) or Lou Gehrig's Disease	○	○
Anorexia or Bulimia	○	○
Antisocial personality disorder	○	○
Asthma	○	○
	Yes	No
Autism Spectrum Disorder	○	○
Avoidant personality disorder	○	○
Bell's Palsy	○	○
Binge Eating Disorder	○	○
Bipolar Disorder	○	○
Bladder Pain or Interstitial Cystitis	○	○
Bladder Stones	○	○
Body-dysmorphic disorder	○	○
Borderline Personality Disorder	○	○
Brain Injury	○	○
	Yes	No
Breast Cancer	○	○
Carpal Tunnel Syndrome	○	○
Cerebral Palsy	○	○
Chronic Fatigue Syndrome	○	○
Chronic Kidney Disease	○	○
Chronic Liver Disease+A39	○	○

	Yes	No
[illegible]	○	○
Chronic Low Back Pain	○	○
Chronic Obstructive Pulmonary Disorder	○	○
Chronic Pelvic Pain	○	○
Cluster Headache Disorder	○	○
	Yes	No
Colorectal Cancer	○	○
Complex Regional Pain Syndrome	○	○
Concussion	○	○
Conversion disorder	○	○
Crohn's Disease	○	○
Dependent personality disorder	○	○
Depersonalization/derealization disorder	○	○
Depression	○	○
Diabetes Type 1	○	○
Diabetes Type 2	○	○
	Yes	No
Diabetic Neuropathy	○	○
Dissociative identity disorder	○	○
Emphysema	○	○
Epilepsy	○	○
Fibromyalgia	○	○
Gambling Addiction or Disorder	○	○
Generalized Anxiety Disorder	○	○
Grave’s Disease	○	○
Heart Attack	○	○
Heart Murmur	○	○
	Yes	No
Hepatitis, alcoholic	○	○
Hepatitis, viral	○	○

	Yes	No
Hepatitis, viral	○	○
Herpes Simplex, Type 1	○	○
Herpes Simplex, Type 2	○	○
Herpes Zoster	○	○
High Blood Pressure	○	○
Histrionic personality disorder	○	○
HIV/AIDS	○	○
Hyperthyroidism	○	○
Hypothyroidism	○	○
	Yes	No
Illness anxiety disorder	○	○
Inflammatory Arthritis	○	○
Inflammatory Bowel Syndrome	○	○
Insomnia	○	○
Kidney Stones	○	○
Liver Cirrhosis	○	○
Lung Cancer	○	○
Lupus	○	○
Lyme Disease	○	○
Migraine Disorder	○	○
	Yes	No
Multiple Chemical Sensitivity	○	○
Multiple Sclerosis	○	○
Muscular Dystrophy	○	○
Narcissistic personality disorder	○	○
Narcolepsy	○	○
Nephritis	○	○
Neurofibromatosis	○	○
Obesity	○	○
Obsessive Compulsive Disorder	○	○
Obsessive-compulsive personality disorder	○	○

	Yes	No
Opiate Addiction	○	○
Other phobia	○	○
Ovarian Cancer	○	○
Pancreatic Cancer	○	○
Panic Disorder	○	○
Paralysis	○	○
Paranoid personality disorder	○	○
Parkinson's Disease	○	○
Peripheral Artery Disorder	○	○
Peripheral neuropathy	○	○
	Yes	No
Postpartum Depression	○	○
Posttraumatic Stress Disorder	○	○
Premenstrual dysphoric disorder	○	○
Prostate Cancer	○	○
Prostatitis	○	○
Radiculopathy	○	○
Restless legs syndrome	○	○
Rheumatoid Arthritis	○	○
Schizoid personality disorder	○	○
Schizophrenia	○	○
	Yes	No
Schizotypal personality disorder	○	○
Sciatica	○	○
Scoliosis	○	○
Seizure	○	○
Skin Cancer	○	○
Social Anxiety Disorder	○	○
Somatoform Disorder	○	○
Spinal Cord Injury, Cervical	○	○

	Yes	No
[illegible]	○	○
Spinal Cord Injury, Lumbar	○	○
Spinal Cord Injury, Thoracic	○	○
	Yes	No
Stimulant Addiction (e.g. methamphetamine or cocaine addiction)	○	○
Stomach Cancer	○	○
Stroke	○	○
Temporomandibular Joint Disorder	○	○
Tongue or Mouth Cancer	○	○
Trichotillomania (hair-pulling disorder)	○	○
Trigeminal Neuralgia	○	○
Tuberculosis	○	○

Have you been hospitalized within the last 6 months?

- ○ Yes
- ○ No

If yes, please explain.

[ ]

Have you had surgery within the last 6 months?

- ○ Yes
- ○ No

If yes, please explain.

[ ]

Are you currently pregnant?

- Yes
- No

If yes, what trimester are you in?

- 1st trimester
- 2nd trimester
- 3rd trimester

Do you have a pacemaker?

- Yes
- No

Do you have an internal defibrillator?

- Yes
- No

Do you currently regularly take any prescription medications?

- Yes
- No

If yes, please list your prescription medications.

Do you currently regularly take any over-the-counter medications?

- Yes
- No

If yes, please list your over-the-counter medications.

Do you currently regularly take vitamins or supplements?

- Yes
- No

If yes, please list your vitamins or supplements.

Do you currently smoke tobacco?

- Yes
- No

How much do you smoke?

- less than 1 pack per week
- less than 1/2 pack per day
- 1 to 1 1/2 packs per day
- 2 to 3 packs per day
- 3 or more packs per day

Please choose the answer "B"

- A
- B
- C

Have you ever smoked tobacco?

- Yes, I quit smoking within the last 6 months.
- Yes, I quit smoking within the last year.
- Yes, I quit smoking in the last 2 years.
- Yes, I quit smoking more than 2 years ago.
- No, I have never smoked.

Do you smoke marijuana?

- Yes
- No

Do you use illicit drugs?

- Yes
- No

Do you drink alcohol?

- Yes
- No

How much do you alcohol do you drink?

- 1 or 2 drinks per month
- 1 or 2 drinks per week
- 3 to 5 drinks per week
- 6 to 12 drinks per week
- 2 to 5 drinks per day
- 6 or more drinks per day

Do you drink caffeinated beverages (such as coffee or tea)?

- Yes
- No

How many caffeinated beverages do you drink?

- 1 or 2 drinks per month
- 1 or 2 drinks per week
- 3 to 5 drinks per week
- 6 to 12 drinks per week
- 1 to 2 drinks per day
- 3 to 5 drinks per day
- 6 or more drinks per day

For the next part of the survey we will ask you questions about your feelings, beliefs and behaviors in relationship to the COVID-19 pandemic which began in the United States about January 20, 2020. Please choose the answer that first comes to you. Some of these questions may be of a personal nature. Please remember that your answers will be kept confidential and data will be kept as secure as possible.

Have you been staying at home to avoid infection from the novel coronavirus or COVID-19?

- Yes
- No

On about what date did you begin social distancing or quarantine?

How many people do you live with in your residence?

How many pets do you have living with you?

About how many people, outside of your immediate household, have you been within 6 feet of in the past week?

About how many people, outside of your immediate household, have you been in physical contact within the past week?

About how often within the last week have you left your place of residence within the last week aside from exercise or walks on your own property?

How often do you wear a mask when you go outside to locations other than your place of residence?

- Always
- Most of the time
- About half the time
- Sometimes
- Never

What type of mask do you wear?

- none
- scarf or bandana
- cloth mask
- medical mask
- N95 respirator

Are you employed in an essential job that you travel to more than twice a week?

- Yes, daily.
- Yes, 3 to 5 times per week.
- Yes, once or twice per week.
- No

Are you able to perform most of your job functions working remotely from home?

- Yes
- No

How worried are you that you will be exposed to the novel coronavirus or COVID-19 at your place of employment?

- Not at all worried
- A little bit worried
- Moderately worried
- Quite a bit worried
- Extremely worried

Do you wear any personal protective equipment while at work?

- N95 respirator
- Medical Mask
- cloth mask
- face shield
- plastic guard

Have you taken any vitamins or medications in an attempt to lower your risk of disease form the novel coronavirus or COVID-19?

- Vitamin C
- Vitamin D
- Multivitamin
- ____ Other supplement
- ____ Over-the-counter medication
- ____ Prescription medication

How often are you exercising per week?

- Never
- once or twice a week
- 3 to 5 times per week
- Daily

How many sides does a square have?

- 2
- 4
- 6
- 11

Have you taken your temperature within the past week?

- No
- once or twice
- 3 to 5 times
- Daily

How often have you had a cough in the past week?

- No
- once or twice
- 3 to 5 times
- Daily
- A few times per day
- Persistent

How often have you been short of breath or had trouble breathing in the past week?

- No
- once or twice
- 3 to 5 times
- Daily
- A few times per day
- Persistent

Have you had a fever in the past week?

- No
- once or twice
- 3 to 5 times
- Daily
- A few times per day
- Persistent

How often have you experienced chills in the past week?

- No
- once or twice
- 3 to 5 times
- Daily
- A few times per day
- Persistent

Have you lost your sense of smell in the last month?

- No
- On one or two days
- Weekly
- Daily
- Persistent

Have you been tested for the novel coronavirus or COVID-19 this past month?

- Yes
- No

Have you tested positive for the novel coronavirus or COVID-19 this past month?

- Yes
- No

Have you spoken with a doctor in a professional capacity (had a consult) about symptoms you believe to be related to the novel coronavirus or COVID-19 this past month?

- No
- Once or twice
- Weekly
- A couple of times per week
- Daily

Whether or not you have been tested for the coronavirus or COVID-19, do you believe that you have contracted the coronavirus or COVID-19 in the past month?

- Definitely yes
- Probably yes
- Probably not
- Definitely not

Do you believe you have been in contact with someone who has the novel coronavirus or COVID-19 in the past week?

- Definitely yes
- Probably yes
- Probably not
- Definitely not

How worried are you that you may contract the coronavirus or COVID-19 in the next month?

- Not at all worried
- A little bit worried
- Moderately worried
- Quite a bit worried
- Extremely worried

How concerned are you to go outside of your place of residence regarding your risk of infection with the novel coronavirus or COVID-19?

- Not at all concerned
- A little bit concerned
- Moderately concerned
- Quite a bit concerned
- Extremely concerned

What year is it?

- 1999
- 2019
- 2020
- 2024

How much news or other COVID-19 related content have you watched each day in the past week?

- None
- One or two hours per week
- 3 to 5 hours per week
- About one hour per day
- 2 to 5 hours a day
- Most hours of the day

Considering any chronic diseases you may have, how concerned are you that you may contract the novel coronavirus or COVID-19 in the next month?

- Not at all concerned or have no chronic diseases
- A little bit concerned
- Moderately concerned
- Quite a bit concerned
- Extremely concerned

Do you believe that you have a higher risk for serious illness from the novel coronavirus or COVID-19?

- No
- Yes, at a slightly increased risk
- Yes, at a moderately increased risk
- Yes, at a severely increased risk

Questions 88 through 93 list difficulties people sometimes have after stressful life events. Please read each item, and then indicate how distressing each difficulty has been for you DURING THE PAST SEVEN DAYS with respect to COVID-19 pandemic, which began in late January 2020. How much were you distressed or bothered by these difficulties?

Other things kept making me think about it.

- Not at all
- A little bit
- Moderately
- Quite a bit
- Extremely

I thought about it when I didn't mean to.

- Not at all
- A little bit
- Moderately
- Quite a bit
- Extremely

I tried not to think about it.

- Not at all
- A little bit
- Moderately
- Quite a bit
- Extremely

I was aware that I still had a lot of feelings about it, but I didn't deal with them.

- Not at all
- A little bit
- Moderately
- Quite a bit
- Extremely

I had trouble concentrating.

- Not at all
- A little bit
- Moderately
- Quite a bit
- Extremely

I felt watchful and on-guard.

- Not at all
- A little bit
- Moderately
- Quite a bit
- Extremely

How many sides does a triangle have?

- 2
- 3
- 5

For the next part of the survey we will ask you questions about your feelings and emotions. Please choose the answer that first comes to you. Some of these questions may be of a personal nature. Please remember that your answers will be kept confidential and data will be kept as secure as possible.

I think that my physical symptoms are signs of a serious illness.

- Never
- Rarely
- Sometimes
- Often
- Very Often

I am very worried about my health

- Never
- Rarely
- Sometimes
- Often
- Very Often

My health concerns hinder me in everyday life

- Never
- Rarely
- Sometimes
- Often
- Very Often

I am convinced that my symptoms are serious

- Never
- Rarely
- Sometimes
- Often
- Very Often

My symptoms scare me

- Never
- Rarely
- Sometimes
- Often
- Very Often

My physical complaints occupy me for most of the day

- Never
- Rarely
- Sometimes
- Often
- Very Often

If you have 3 apples and your friend gives you 4 apples, how many apples do you have?

- 3
- 4
- 7
- 9
- 12

Others tell me that my physical problems are not serious

- Never
- Rarely
- Sometimes
- Often
- Very Often

I'm worried that my physical complaints will never stop

- Never
- Rarely
- Sometimes
- Often
- Very Often

My worries about my health take my energy

- Never
- Rarely
- Sometimes
- Often
- Very Often

I think that doctors do not take my physical complaints seriously

- Never
- Rarely
- Sometimes
- Often
- Very Often

I am worried that my physical symptoms will continue into the future

- Never
- Rarely
- Sometimes
- Often
- Very Often

Due to my physical complaints, I have poor concentration on other things

- Never
- Rarely
- Sometimes
- Often
- Very Often

During the past 7 days, how much have you been bothered by any of the following problems?

	Not at all	A little bit	Somewhat	Quite a bit	Very much
Stomach or bowel problems	○	○	○	○	○
Back pain	○	○	○	○	○
Pain in your arms, legs, or joints	○	○	○	○	○
Headaches	○	○	○	○	○
Chest pain or shortness of breath	○	○	○	○	○
Dizziness	○	○	○	○	○
Feeling tired or having low energy	○	○	○	○	○
Trouble sleeping	○	○	○	○	○

Over the last 2 weeks how often have you been bothered by any of the following problems:

	Not at all	Several days	More than half of the days	Nearly every day
Feeling nervous, anxious or on edge	○	○	○	○
Not being able to stop or control worrying	○	○	○	○
Worrying too much about different things	○	○	○	○
Trouble relaxing	○	○	○	○
Being so restless that it is hard to sit still	○	○	○	○
Becoming easily annoyed or irritable	○	○	○	○
Feeling afraid as if something awful might happen	○	○	○	○

How difficult have these problems made it for you to do your work, take care of things at home, or get along with other people?

- Not difficult at all
- Somewhat difficult
- Very difficult
- Extremely difficult

Over the last 2 weeks how often have you been bothered by any of the following problems:

	Not at all	Several days	More than half of the days	Nearly every day
Little interest or pleasure in doing things	○	○	○	○
Feeling down, depressed or hopeless	○	○	○	○
Trouble falling or staying asleep, or sleeping too much	○	○	○	○
Feeling tired or having little energy	○	○	○	○
Poor appetite or overeating	○	○	○	○
Feeling bad about yourself - or that you are a failure or have let yourself or your family down	○	○	○	○
Trouble concentrating on things, such as reading the newspaper or watching television	○	○	○	○
Moving or speaking so slowly that other people could have noticed. Or the opposite - being so figety or restless that you have been moving around a lot more than usual.	○	○	○	○
Thoughts that you would be better off dead, or of hurting yourself	○	○	○	○

How difficult have these problems made it for you to do your work, take care of things at home, or get along with other people?

- Not difficult at all
- Somewhat difficult
- Very difficult
- Extremely difficult

4 + 5 =

- 5
- 6
- 9
- 12

The questions from 108 to 121 ask you about your feelings and thoughts during the last month. In each case, you will be asked to indicate how often you felt or thought a certain way. Although some of the questions are similar, there are differences between them and you should treat each one as a separate question. The best approach is to answer each question fairly quickly. That is, don't try to count up the number of times you felt a particular way, but rather indicate the alternative that seems like a reasonable estimate.

In the last month, how often have you been upset because of something that happened unexpectedly?

- Never
- Almost never
- Sometimes
- Fairly often
- Very often

In the last month, how often have you felt that you were unable to control the important things in your life?

- Never
- Almost never
- Sometimes
- Fairly often
- Very often

In the last month, how often have you felt nervous and "stressed"?

- Never
- Almost never
- Sometimes
- Fairly often
- Very often

In the last month, how often have you dealt successfully with irritating life hassles?

- Never
- Almost never
- Sometimes
- Fairly often
- Very often

In the last month, how often have you felt that you were effectively coping with important changes that were occurring in your life?

- Never
- Almost never
- Sometimes
- Fairly often
- Very often

In the last month, how often have you felt confident about your ability to handle your personal problems?

- Never
- Almost never
- Sometimes
- Fairly often
- Very often

In the last month, how often have you felt that things were going your way?

- Never
- Almost never
- Sometimes
- Fairly often
- Very often

In the last month, how often have you found that you could not cope with all the things that you had to do?

- Never
- Almost never
- Sometimes
- Fairly often
- Very often

In the last month, how often have you been able to control irritations in your life?

- Never
- Almost never
- Sometimes
- Fairly often
- Very often

In the last month, how often have you felt that you were on top of things?

- Never
- Almost never
- Sometimes
- Fairly often
- Very often

In the last month, how often have you been angered because of things that happened that were outside of your control?

- Never
- Almost never
- Sometimes
- Fairly often
- Very often

In the last month, how often have you found yourself thinking about things that you have to accomplish?

- Never
- Almost never
- Sometimes
- Fairly often
- Very often

What planet do you live on?

- Mars
- Venus
- Mercury
- Earth

In the last month, how often have you been able to control the way you spend your time?

- Never
- Almost never
- Sometimes
- Fairly often
- Very often

In the last month, how often have you felt difficulties were piling up so high that you could not overcome them?

- Never
- Almost never
- Sometimes
- Fairly often
- Very often

Below is a list of problems and complaints that people sometimes have. Please read each one carefully and choose the number that best describes how much you were bothered by that problem during the past week. Please choose only ONE.

FOR THE PAST WEEK, HOW MUCH WERE YOU BOTHERED BY:

	Not at all	A little bit	Moderately	Quite a bit	Extremely
Headaches	○	○	○	○	○
Nervousness or shakiness inside	○	○	○	○	○
Unwanted thoughts, words, or ideas that won't leave your mind	○	○	○	○	○
Faintness or dizziness	○	○	○	○	○
Loss of sexual interest or pleasure	○	○	○	○	○
Feeling critical of others	○	○	○	○	○
The idea that someone else can control your thoughts	○	○	○	○	○
Feeling others are to blame for most of your troubles	○	○	○	○	○
Trouble remembering things	○	○	○	○	○
	Not at all	A little bit	Moderately	Quite a bit	Extremely
Worried about sloppiness or carelessness	○	○	○	○	○
Feeling easily annoyed or irritated	○	○	○	○	○
Pains in heart or chest	○	○	○	○	○
Feeling afraid in open spaces or on the streets	○	○	○	○	○
Feeling low in energy or slowed down	○	○	○	○	○
Thoughts of ending your life	○	○	○	○	○
Hearing words that others do not hear	○	○	○	○	○
Trembling	○	○	○	○	○

Feeling that most people cannot be trusted	○	○	○	○	○

	Not at all	A little bit	Moderately	Quite a bit	Extremely
Poor appetite	○	○	○	○	○
Crying easily	○	○	○	○	○
Feeling shy or uneasy with the opposite sex	○	○	○	○	○
Feeling of being trapped or caught	○	○	○	○	○
Suddenly scared for no reason	○	○	○	○	○
Temper outbursts that you could not control	○	○	○	○	○
Feeling afraid to go out of your house alone	○	○	○	○	○
Blaming yourself for things	○	○	○	○	○
Pains in lower back	○	○	○	○	○

	Not at all	A little bit	Moderately	Quite a bit	Extremely
Feeling blocked in getting things done	○	○	○	○	○
Feeling lonely	○	○	○	○	○
Feeling blue	○	○	○	○	○
Worrying too much about things	○	○	○	○	○
Feeling no interest in things	○	○	○	○	○
Feeling fearful	○	○	○	○	○
Your feelings being easily hurt	○	○	○	○	○
Other people being aware of your private thoughts	○	○	○	○	○
Feeling others do not understand you or are unsympathetic	○	○	○	○	○

[illegible]

	Not at all	A little bit	Moderately	Quite a bit	Extremely
Feeling that people are unfriendly or dislike you					
Having to do things very slowly to insure correctness					
Heart pounding or racing					
Nausea or upset stomach					
Feeling inferior to others					
Soreness of your muscles					
Feeling that you are watched or talked about by others					
Trouble falling asleep					
Having to check and double-check what you do					

	Not at all	A little bit	Moderately	Quite a bit	Extremely
Difficulty making decisions					
Feeling afraid to travel on buses, subways, or trains					
Trouble getting your breath					
Hot or cold spells					
Having to avoid certain things, places, or activities because they frighten you					
Your mind going blank					
Numbness or tingling in parts of your body					
A lump in your throat					

A lump in your throat	○	○	○	○	○
Feeling hopeless about the future	○	○	○	○	○

	Not at all	A little bit	Moderately	Quite a bit	Extremely
Trouble concentrating	○	○	○	○	○
Feeling weak in parts of your body	○	○	○	○	○
Feeling tense or keyed up	○	○	○	○	○
Heavy feelings in your arms or legs	○	○	○	○	○
Thoughts of death or dying	○	○	○	○	○
Overeating	○	○	○	○	○
Feeling uneasy when people are watching or talking about you	○	○	○	○	○
Having thoughts that are not your own	○	○	○	○	○
Having urges to beat, injure, or harm someone	○	○	○	○	○

	Not at all	A little bit	Moderately	Quite a bit	Extremely
Awakening in the early morning	○	○	○	○	○
Having to repeat the same actions such as touching, counting, washing	○	○	○	○	○
Sleep that is restless or disturbed	○	○	○	○	○
Having urges to break or smash things	○	○	○	○	○
Having ideas or beliefs that others do not share	○	○	○	○	○
Feeling very self-conscious with others	○	○	○	○	○
Feeling uneasy in crowds, such as					

	Not at all	A little bit	Moderately	Quite a bit	Extremely
crowds, such as shopping or at a movie	○	○	○	○	○
Feeling everything is an effort	○	○	○	○	○
Spells of terror or panic	○	○	○	○	○

	Not at all	A little bit	Moderately	Quite a bit	Extremely
Feeling uncomfortable about eating or drinking in public	○	○	○	○	○
Getting into frequent arguments	○	○	○	○	○
Feeling nervous when you are left alone	○	○	○	○	○
Others not giving you proper credit for your achievements	○	○	○	○	○
Feeling lonely even when you are with people	○	○	○	○	○
Feeling so restless you couldn't sit still	○	○	○	○	○
Feelings of worthlessness	○	○	○	○	○
Feeling that familiar things are strange or unreal	○	○	○	○	○
Shouting or throwing things	○	○	○	○	○

	Not at all	A little bit	Moderately	Quite a bit	Extremely
Feeling afraid you will faint in public	○	○	○	○	○
Feeling that people will take advantage of you if you let them	○	○	○	○	○
Having thoughts about sex that bother you a lot	○	○	○	○	○
The idea that you should be punished	○	○	○	○	○

should be punished for your sins	◯	◯	◯	◯	◯
Feeling pushed to get things done	◯	◯	◯	◯	◯
The idea that something serious is wrong with your body	◯	◯	◯	◯	◯
Never feeling close to another person	◯	◯	◯	◯	◯
Feelings of guilt	◯	◯	◯	◯	◯
The idea that something is wrong with your mind	◯	◯	◯	◯	◯

It is important for me not to appear nervous.

- ◯ None or a little
- ◯ A little
- ◯ Some
- ◯ Much
- ◯ Very much

When I cannot keep my mind on a task, I worry that I might be going crazy.

- ◯ None or a little
- ◯ A little
- ◯ Some
- ◯ Much
- ◯ Very much

It scares me when I feel “shaky” (trembling).

- None or a little
- A little
- Some
- Much
- Very much

It scares me when I feel faint.

- None or a little
- A little
- Some
- Much
- Very much

It is important for me to stay in control of my emotions.

- None or a little
- A little
- Some
- Much
- Very much

It scares me when my heart beats rapidly.

- None or a little
- A little
- Some
- Much
- Very much

It embarrasses me when my stomach growls.

- None or a little
- A little
- Some
- Much
- Very much

It scares me when I am nauseous.

- None or a little
- A little
- Some
- Much
- Very much

When I notice that my heart is beating rapidly, I worry I might have a heart attack.

- None or a little
- A little
- Some
- Much
- Very much

1 + 2 =

- 3
- 7
- 11

It scares me when I become short of breath.

- None or a little
- A little
- Some
- Much
- Very much

When my stomach is upset, I worry that I might be seriously ill.

- None or a little
- A little
- Some
- Much
- Very much

It scares me when I am unable to keep my mind on a task.

- None or a little
- A little
- Some
- Much
- Very much

Other people notice when I feel shaky.

- None or a little
- A little
- Some
- Much
- Very much

Unusual body sensations scare me.

- None or a little
- A little
- Some
- Much
- Very much

When I am nervous, I worry that I might be mentally ill.

- None or a little
- A little
- Some
- Much
- Very much

It scares me when I am nervous.

- None or a little
- A little
- Some
- Much
- Very much

Mark one response for each item and do not skip any items.

	Never or almost never have	Less than 3 or 4 times per year	Every month or so	Every week or so	More than once every week
Eyes water	○	○	○	○	○
Itchy eyes or skin	○	○	○	○	○
Ringing in ears	○	○	○	○	○
Temporary deafness or hard of hearing	○	○	○	○	○
Lump in throat	○	○	○	○	○
Choking sensations	○	○	○	○	○
Sneezing spells	○	○	○	○	○
Running nose	○	○	○	○	○

	Never or almost never have	Less than 3 or 4 times per year	Every month or so	Every week or so	More than once every week
Congested nose	○	○	○	○	○
Bleeding nose	○	○	○	○	○
Asthma or wheezing	○	○	○	○	○
Coughing	○	○	○	○	○
Out of breath	○	○	○	○	○
Swollen ankles	○	○	○	○	○
Chest pains	○	○	○	○	○
Racing heart	○	○	○	○	○

	Never or almost never have	Less than 3 or 4 times per year	Every month or so	Every week or so	More than once every week
Cold hands or feet even in hot weather	○	○	○	○	○
Leg cramps	○	○	○	○	○
Insomnia or difficulty sleeping	○	○	○	○	○
Toothaches	○	○	○	○	○
Upset stomach	○	○	○	○	○
Indigestion	○	○	○	○	○

[illegible]	○	○	○	○	○
Heartburn or gas	○	○	○	○	○
Abdominal pain	○	○	○	○	○
	Never or almost never have	Less than 3 or 4 times per year	Every month or so	Every week or so	More than once every week
Diarrhea	○	○	○	○	○
Constipation	○	○	○	○	○
Hemorrhoids	○	○	○	○	○
Swollen joints	○	○	○	○	○
Stiff or sore muscles	○	○	○	○	○
Back pains	○	○	○	○	○
Sensitive or tender skin	○	○	○	○	○
Face flushes	○	○	○	○	○
	Never or almost never have	Less than 3 or 4 times per year	Every month or so	Every week or so	More than once every week
Tightness in chest	○	○	○	○	○
Skin breaks out in rash	○	○	○	○	○
Acne or pimples on face	○	○	○	○	○
Acne / pimples other than face	○	○	○	○	○
Boils	○	○	○	○	○
Sweat even in cold weather	○	○	○	○	○
Strong reaction to insect bites	○	○	○	○	○
Headaches	○	○	○	○	○
	Never or almost never have	Less than 3 or 4 times per year	Every month or so	Every week or so	More than once every week
Feeling pressure in head	○	○	○	○	○
Hot flashes	○	○	○	○	○

Chills	○	○	○	○	○
Dizziness	○	○	○	○	○
Feel faint	○	○	○	○	○
Numbness or tingling in any part of body	○	○	○	○	○
Twitching of eyelid	○	○	○	○	○
Twitching other than eyelid	○	○	○	○	○
	Never or almost never have	Less than 3 or 4 times per year	Every month or so	Every week or so	More than once every week
Hands tremble or shake	○	○	○	○	○
Stiff joints	○	○	○	○	○
Sore muscles	○	○	○	○	○
Sore throat	○	○	○	○	○
Sunburn	○	○	○	○	○
Nausea	○	○	○	○	○

How Accurately Can You Describe Yourself?

Please use this list of common human traits to describe yourself as accurately as possible. Describe yourself as you see yourself at the present time, not as you wish to be in the future. Describe yourself as you are generally or typically, as compared with other persons you know of the same sex and of roughly your same age.

Before each trait, please write a number indicating how accurately that trait describes you, using the following rating scale:

		**Inaccurate**					**Accurate**		
	Extremely Inaccurate	Very	Quite	Slightly	Neither	Slightly	Quite	Very	Extremely Accurate
Self-pitying	○	○	○	○	○	○	○	○	○
High-strung	○	○	○	○	○	○	○	○	○
Imaginative	○	○	○	○	○	○	○	○	○
Unimaginative	○	○	○	○	○	○	○	○	○
Unadventurous	○	○	○	○	○	○	○	○	○
Relaxed	○	○	○	○	○	○	○	○	○

Assertive	○	○	○	○	○	○	○	○	○
Considerate	○	○	○	○	○	○	○	○	○
Extraverted	○	○	○	○	○	○	○	○	○
Bashful	○	○	○	○	○	○	○	○	○

	Extremely Inaccurate	Very	Quite	Slightly	Neither	Slightly	Quite	Very	Extremely Accurate
Fretful	○	○	○	○	○	○	○	○	○
Reserved	○	○	○	○	○	○	○	○	○
Agreeable	○	○	○	○	○	○	○	○	○
Selfish	○	○	○	○	○	○	○	○	○
Steady	○	○	○	○	○	○	○	○	○
Timid	○	○	○	○	○	○	○	○	○
Introspective	○	○	○	○	○	○	○	○	○
Uncreative	○	○	○	○	○	○	○	○	○
Untalkative	○	○	○	○	○	○	○	○	○
Practical	○	○	○	○	○	○	○	○	○

	Extremely Inaccurate	Very	Quite	Slightly	Neither	Slightly	Quite	Very	Extremely Accurate
Anxious	○	○	○	○	○	○	○	○	○
Harsh	○	○	○	○	○	○	○	○	○
Warm	○	○	○	○	○	○	○	○	○
Thorough	○	○	○	○	○	○	○	○	○
Complex	○	○	○	○	○	○	○	○	○
Bold	○	○	○	○	○	○	○	○	○
Systematic	○	○	○	○	○	○	○	○	○
Unintellectual	○	○	○	○	○	○	○	○	○
Unsystematic	○	○	○	○	○	○	○	○	○
Impractical	○	○	○	○	○	○	○	○	○

	Extremely Inaccurate	Very	Quite	Slightly	Neither	Slightly	Quite	Very	Extremely Accurate
Haphazard	○	○	○	○	○	○	○	○	○
Innovative	○	○	○	○	○	○	○	○	○
Shy	○	○	○	○	○	○	○	○	○

Helpful	○	○	○	○	○	○	○	○	○
Emotional	○	○	○	○	○	○	○	○	○
Insecure	○	○	○	○	○	○	○	○	○
Uninquisitive	○	○	○	○	○	○	○	○	○
Cold	○	○	○	○	○	○	○	○	○
Sympathetic	○	○	○	○	○	○	○	○	○
Talkative	○	○	○	○	○	○	○	○	○

	Extremely Inaccurate	Very	Quite	Slightly	Neither	Slightly	Quite	Very	Extremely Accurate
Cooperative	○	○	○	○	○	○	○	○	○
Unenvious	○	○	○	○	○	○	○	○	○
Organized	○	○	○	○	○	○	○	○	○
Generous	○	○	○	○	○	○	○	○	○
Uncooperative	○	○	○	○	○	○	○	○	○
Negligent	○	○	○	○	○	○	○	○	○
Verbal	○	○	○	○	○	○	○	○	○
Inefficient	○	○	○	○	○	○	○	○	○
Efficient	○	○	○	○	○	○	○	○	○
Unemotional	○	○	○	○	○	○	○	○	○

	Extremely Inaccurate	Very	Quite	Slightly	Neither	Slightly	Quite	Very	Extremely Accurate
Bright	○	○	○	○	○	○	○	○	○
Rude	○	○	○	○	○	○	○	○	○
Nervous	○	○	○	○	○	○	○	○	○
Imperceptive	○	○	○	○	○	○	○	○	○
Uncharitable	○	○	○	○	○	○	○	○	○
Envious	○	○	○	○	○	○	○	○	○
Kind	○	○	○	○	○	○	○	○	○
Unkind	○	○	○	○	○	○	○	○	○
Quiet	○	○	○	○	○	○	○	○	○
Unreflective	○	○	○	○	○	○	○	○	○

	Extremely Inaccurate	Very	Quite	Slightly	Neither	Slightly	Quite	Very	Extremely Accurate
Careful	○	○	○	○	○	○	○	○	○

Careful									
Conscientious									
Undemanding									
Disorganized									
Inhibited									
Simple									
Unintelligent									
Artistic									
Unrestrained									
Pleasant									

	Extremely Inaccurate	Very	Quite	Slightly	Neither	Slightly	Quite	Very	Extremely Accurate
Sloppy									
Imperturbable									
Energetic									
Daring									
Introverted									
Unsympathetic									
Demanding									
Philosophical									
Irritable									
Moody									

	Extremely Inaccurate	Very	Quite	Slightly	Neither	Slightly	Quite	Very	Extremely Accurate
Prompt									
Neat									
Inconsistent									
Unexcitable									
Careless									
Unsophisticated									
Undependable									
Deep									
Distrustful									
[illegible]									

The next 17 questions (numbers 153 to 170) ask you to imagine yourself in certain situations. You should then decide if these situations would be painful for you and if yes, how painful they would be. Let 0 stand for no pain; 1 is an only just noticeable pain and 10 the most severe pain that you

	Extremely Inaccurate	Very	Quite	Slightly	Neither	Slightly	Quite	Very	Extremely Accurate
Touchy	○	○	○	○	○	○	○	○	○
Intellectual	○	○	○	○	○	○	○	○	○
Withdrawn	○	○	○	○	○	○	○	○	○
Trustful	○	○	○	○	○	○	○	○	○
Fearful	○	○	○	○	○	○	○	○	○
Active	○	○	○	○	○	○	○	○	○
Temperamental	○	○	○	○	○	○	○	○	○
Vigorous	○	○	○	○	○	○	○	○	○
Jealous	○	○	○	○	○	○	○	○	○
Shallow	○	○	○	○	○	○	○	○	○
Creative	○	○	○	○	○	○	○	○	○

can imagine or consider possible. Please mark the scale with a cross on the number that is most true for you. Keep in mind that there are no “right” or “wrong” answers; only your personal assessment of the situation counts.

Please try as much as possible not to allow your fear or aversion of the imagined situations affect your assessment of painfulness.

Imagine you bump your shin badly on a hard edge, for example, on the edge of a glass coffee table.

How painful would that be for you?

Not at all painful — Most severe pain imaginable

0 ○ 1 ○ 2 ○ 3 ○ 4 ○ 5 ○ 6 ○ 7 ○ 8 ○ 9 ○ 10 ○

Imagine you burn your tongue on a very hot drink.

How painful would that be for you?

Not at all painful — Most severe pain imaginable

0 ○ 1 ○ 2 ○ 3 ○ 4 ○ 5 ○ 6 ○ 7 ○ 8 ○ 9 ○ 10 ○

Imagine your muscles are slightly sore as the result of physical activity.

How painful would that be for you?

Not at all painful — Most severe pain imaginable

0 ○ 1 ○ 2 ○ 3 ○ 4 ○ 5 ○ 6 ○ 7 ○ 8 ○ 9 ○ 10 ○

What is the disease that is at pandemic levels around the world right now?

- ○ influenza
- ○ COVID-19
- ○ tuberculosis
- ○ polio

Imagine you trap your finger in a drawer.

How painful would that be for you?

Not at all painful | Most severe pain imaginable

0○ 1○ 2○ 3○ 4○ 5○ 6○ 7○ 8○ 9○ 10○

Imagine you take a shower with lukewarm water.

How painful would that be for you?

Not at all painful | Most severe pain imaginable

0○ 1○ 2○ 3○ 4○ 5○ 6○ 7○ 8○ 9○ 10○

Imagine you have mild sunburn on your shoulders.

How painful would that be for you?

Not at all painful | Most severe pain imaginable

0○ 1○ 2○ 3○ 4○ 5○ 6○ 7○ 8○ 9○ 10○

Imagine you grazed your knee falling off your bicycle.

How painful would that be for you?

Not at all painful | Most severe pain imaginable

0○ 1○ 2○ 3○ 4○ 5○ 6○ 7○ 8○ 9○ 10○

Imagine you accidentally bite your tongue or cheek badly while eating.

How painful would that be for you?

Not at all painful | Most severe pain imaginable

0○ 1○ 2○ 3○ 4○ 5○ 6○ 7○ 8○ 9○ 10○

Imagine walking across a cool tiled floor with bare feet.

How painful would that be for you?

Not at all painful | Most severe pain imaginable

0○ 1○ 2○ 3○ 4○ 5○ 6○ 7○ 8○ 9○ 10○

Imagine you have a minor cut on your finger and inadvertently get lemon juice in the wound.
How painful would that be for you?

Not at all painful Most severe pain imaginable
0 1 2 3 4 5 6 7 8 9 10

Imagine you prick your fingertip on the thorn of a rose.
How painful would that be for you?

Not at all painful Most severe pain imaginable
0 1 2 3 4 5 6 7 8 9 10

Imagine you stick your bare hands in the snow for a couple of minutes or bring your hands in contact with snow for some time, for example, while making snowballs.
How painful would that be for you?

Not at all painful Most severe pain imaginable
0 1 2 3 4 5 6 7 8 9 10

Imagine you shake hands with someone who has a normal grip.
How painful would that be for you?

Not at all painful Most severe pain imaginable
0 1 2 3 4 5 6 7 8 9 10

Imagine you shake hands with someone who has a very strong grip.
How painful would that be for you?

Not at all painful Most severe pain imaginable
0 1 2 3 4 5 6 7 8 9 10

Imagine you pick up a hot pot by inadvertently grabbing its equally hot handles.
How painful would that be for you?

Not at all painful Most severe pain imaginable
0 1 2 3 4 5 6 7 8 9 10

Imagine you are wearing sandals and someone with heavy boots steps on your foot. How painful would that be for you?

Not at all painful — Most severe pain imaginable

0 ○ 1 ○ 2 ○ 3 ○ 4 ○ 5 ○ 6 ○ 7 ○ 8 ○ 9 ○ 10 ○

Imagine you bump your elbow on the edge of a table (“funny bone”). How painful would that be for you?

Not at all painful — Most severe pain imaginable

0 ○ 1 ○ 2 ○ 3 ○ 4 ○ 5 ○ 6 ○ 7 ○ 8 ○ 9 ○ 10 ○

The following questions (172 to 183) relate to your usual sleep habits during the past month only. Your answers should indicate the most accurate reply for the majority of days and nights in the past month.

During the past month, when have you usually gone to bed at night?

[ ]

During the past month, how long (in minutes) has it usually take you to fall asleep each night?

[ ]

During the past month, when have you usually gotten up in the morning?

[ ]

During the past month, how many hours of actual sleep did you get at night? (This may be different than the number of hours you spend in bed.)

[ ]

During the past month, how often have you had trouble sleeping because you...

	Not during the past month	Less than once a week	Once or twice a week	Three or more times a week
Cannot get to sleep within 30 minutes	○	○	○	○
Wake up in the middle of the night or early morning	○	○	○	○
Have to get up to use the bathroom	○	○	○	○
Cannot breathe comfortably	○	○	○	○
Cough or snore loudly	○	○	○	○
Feel too cold	○	○	○	○
Feel too hot	○	○	○	○
Have bad dreams	○	○	○	○
Have pain	○	○	○	○

During the past month, how would you rate your sleep quality overall?

- ○ Very good
- ○ Fairly good
- ○ Fairly bad
- ○ Very bad

Which of the following things is alive?

- ○ rock
- ○ steel
- ○ rubber
- ○ bird

During the past month, how often have you taken medicine (prescribed or “over the counter”) to help you sleep?

- Not during the past month
- Less than once a week
- Once or twice a week
- Three or more times a week

During the past month, how often have you had trouble staying awake while driving, eating meals, or engaging in social activity?

- Not during the past month
- Less than once a week
- Once or twice a week
- Three of more times a week

During the past month, how much of a problem has it been for you to keep up enough enthusiasm to get things done?

- No problem at all
- Only a very slight problem
- Somewhat of a problem
- A very big problem

Do you have a bed partner or roommate?

- No bed partner or roommate
- Partner/roommate in other room
- Partner in same room, but not same bed
- Partner in same bed

If you have a roommate or bed partner, ask him/her how often in the past month you have had...

	Not during the past month	Less than once a week	Once or twice a week	Three or more times a week
Loud snoring	○	○	○	○
Long pauses between breaths while asleep	○	○	○	○
Legs twitching or jerking while you sleep	○	○	○	○
Episodes of disorientation or confusion during sleep	○	○	○	○

The final section of this survey includes general questions regarding you health and physical abilities.

In general, would you say your health is:

- ○ Excellent
- ○ Very good
- ○ Good
- ○ Fair
- ○ Poor

Compared to one year ago, how would you rate your health in general now?

- ○ Much better now than one year ago
- ○ Somewhat better now than one year ago
- ○ About the same
- ○ Somewhat worse now than one year ago
- ○ Much worse now than one year ago

Does your health now limit you in these activities? If so, how much?

	Yes, limited a lot	Yes, limited a little	No, not limited at all
Vigorous activities, such as running, lifting heavy objects, participating in strenuous sports	○	○	○
Moderate activities, such as moving a table, pushing a vacuum cleaner, bowling, or playing golf	○	○	○
Lifting or carrying groceries	○	○	○
Climbing several flights of stairs	○	○	○
Climbing one flight of stairs	○	○	○
Bending, kneeling, or stooping	○	○	○
Walking more than a mile	○	○	○
Walking several blocks	○	○	○
Walking one block	○	○	○
Bathing or dressing yourself	○	○	○

During the past 4 weeks, have you had any of the following problems with your work or other regular daily activities as a result of your physical health?

	Yes	No
Cut down the amount of time you spent on work or other activities	○	○
Accomplished less than you would like	○	○
Were limited in the kind of work or other activities	○	○
Had difficulty performing the work or other activities (for example, it took extra effort)	○	○

Which of the following objects would be best to write with?

- ○ cooking pot
- ○ pen
- ○ license plate

During the past 4 weeks, have you had any of the following problems with your work or other regular daily activities as a result of any emotional problems (such as feeling depressed or anxious)?

	Yes	No
Cut down the amount of time you spent on work or other activities	○	○
Accomplished less than you would like	○	○
Didn't do work or other activities as carefully as usual	○	○

During the past 4 weeks, to what extent has your physical health or emotional problems interfered with your normal social activities with family, friends, neighbors, or groups?

- Not at all
- Slightly
- Moderately
- Quite a bit
- Extremely

How much bodily pain have you had during the past 4 weeks?

- None
- Very mild
- Mild
- Moderate
- Severe
- Very severe

During the past 4 weeks, how much did pain interfere with your normal work (including both work outside the home and housework)?

- Not at all
- A little bit
- Moderately
- Quite a bit
- Extremely

These questions are about how you feel and how things have been with you during the past 4 weeks. For each question, please give the one answer that comes closest to the way you have been feeling.

How much of the time during the past 4 weeks...

	All of the time	Most of the time	A good bit of the time	Some of the time	A little of the time	None of the time
Did you feel full of pep?	◯	◯	◯	◯	◯	◯
Have you been a very nervous person?	◯	◯	◯	◯	◯	◯
Have you felt so down in the dumps that nothing could cheer you up?	◯	◯	◯	◯	◯	◯
Have you felt calm and peaceful?	◯	◯	◯	◯	◯	◯
Did you have a lot of energy?	◯	◯	◯	◯	◯	◯
Have you felt downhearted and blue?	◯	◯	◯	◯	◯	◯
Did you feel worn out?	◯	◯	◯	◯	◯	◯
Have you been a happy person?	◯	◯	◯	◯	◯	◯
Did you feel tired?	◯	◯	◯	◯	◯	◯

During the past 4 weeks, how much of the time has your physical health or emotional problems interfered with your social activities (like visiting with friends, relatives, etc.)?

- ◯ All of the time
- ◯ Most of the time
- ◯ Some of the time
- ◯ A little of the time
- ◯ None of the time

How TRUE or FALSE is each of the following statements for you.

	Definitely True	Mostly true	Don't know	Mostly false	Definitely false
I seem to get sick a little easier than other people	○	○	○	○	○
I am as healthy as anybody I know	○	○	○	○	○
I expect my health to get worse	○	○	○	○	○
My health is excellent	○	○	○	○	○

Powered by Qualtrics
