# Supplementary figures and images for: A Cross-Sectional Time Course of COVID-19 Related Worry, Perceived Stress, and General Anxiety in the Context of Post-Traumatic Stress Disorder-like Symptomatology
Source: Int J Environ Res Public Health. 2022 Jun 11;19(12):7178. doi: 10.3390/ijerph19127178 (PMC9222603; doi:10.3390/ijerph19127178)

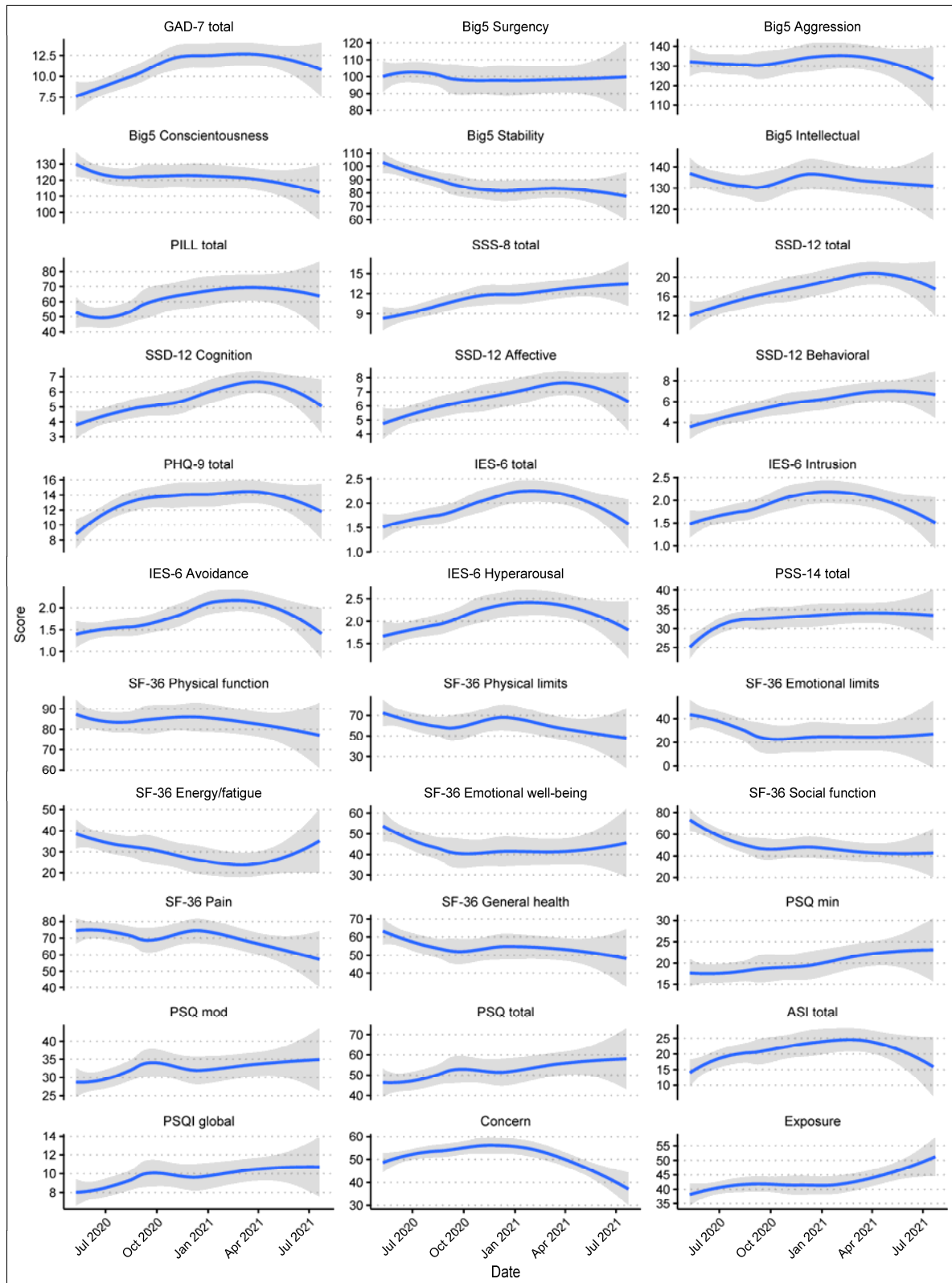

Supplementary\_Figure S1. Complete set of timeline plots for all measure.

Supplement: Supplementary file 1 [file ijerph-19-07178-s001.zip › Supplementary_Figure S1. Complete set of timeline plots for all measure.pdf]
